# The effect of syntactic and semantic information on word grounding through visual perception

**DOI:** 10.3389/frai.2026.1803426

**Published:** 2026-09-22

**Authors:** Saima Shaukat, Amir Aly, Anouar Chibani, Thomas Wennekers, Angelo Cangelosi

**Affiliations:** 1 School of Computing, Engineering, and Mathematics, University of Plymouth, Plymouth, United Kingdom; 2 School of Computer Science, University of Manchester, Manchester, United Kingdom

**Keywords:** Bayesian inference, CLEVR dataset, language models, semantic embeddings, syntactic features, word grounding

## Abstract

**Introduction:**

Word grounding refers to the ability of agents to associate linguistic terms with the perceptual concepts they represent, such as color, shape, and spatial features. This study quantitatively evaluates how syntactic and semantic information affects word-grounding performance.

**Methods:**

We evaluated five configurations within a generative multimodal Bayesian framework. The baseline grounds words using perceptual cues, while the additional configurations independently incorporate word-position indices, part-of-speech tags, FastText embeddings, and transformer-based contextual embeddings. The models were evaluated using a CLEVR-derived 3D scene dataset and synonym-substituted descriptions.

**Results:**

Linguistic information improved grounding accuracy across the Color, Geometry, and Spatial Relation modalities. Contextual embeddings produced a 53.8% absolute improvement in object-grounding accuracy over the baseline. Only models incorporating semantic embeddings generalized effectively to synonym-substituted descriptions without retraining.

**Discussion:**

These findings demonstrate the contribution of linguistic structure and semantic representations to perceptual alignment and provide empirical foundations for developing language-grounding systems.

## 1 Introduction

Enabling agents to link language to the physical world—a process humans perform naturally and effortlessly—remains a central challenge for artificial intelligence. While humans naturally associate words such as *grab* with an action or *sphere* with a rounded object, artificial systems continue to struggle with bridging the gap between abstract linguistic symbols and their real-world perceptual referents. This process is known as *word grounding* (Steels, 2008). It underpins intelligent behavior in dynamic, multimodal settings where language and perception must be tightly integrated. For instance, given the instruction “*pick up the red ball*,” a system must accurately map the word *ball* to the correct visual object based on its shape and appearance, and identify the one with the specified red color among multiple candidates. Figure 1 illustrates this multimodal alignment process.

**Figure 1:**
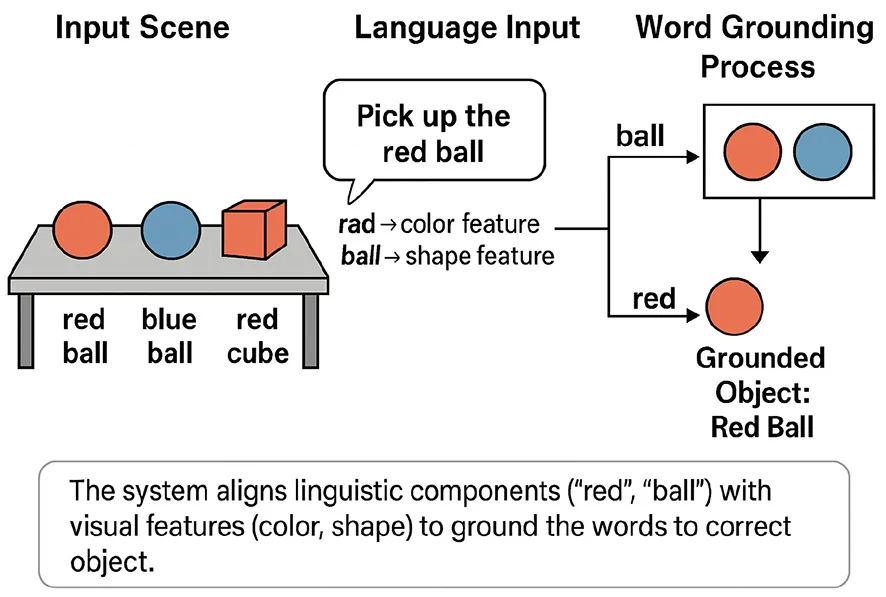
Illustration of the word grounding process.

Early research explored word grounding through symbolic models and interactive robot learning frameworks that established object-word associations through interaction (Roy, 2005, 2002). The symbol grounding problem, introduced by Harnad (1990), highlights the necessity of anchoring linguistic symbols to perceptual and sensorimotor representations. Subsequent advances in visual grounding (Sasaki et al., 2024; Roy et al., 2003; Thomason et al., 2022) have significantly improved the integration of language and perception; yet, a persistent challenge remains: generalizing to unseen synonym substitutions at test time without retraining the grounding model. For example, users may refer to the same object using different lexical forms, such as “automobile” instead of “car,” and a grounding system should ideally handle such substitutions without retraining.

Most existing multimodal grounding approaches rely on direct mappings between observed word forms and visual entities. While effective for seen vocabularies, these methods struggle to generalize when encountering synonyms or unseen linguistic expressions. To overcome this limitation, enriching linguistic representations with additional syntactic and semantic information has emerged as a promising direction. Syntactic cues, such as word position indices and part-of-speech (POS) tags, provide structural information about word roles and contextual positioning, while semantic embeddings derived from pre-trained models capture lexical similarity in continuous vector spaces, enabling models to reason about unseen words based on their semantic proximity.

This paper investigates how different layers of linguistic information—ranging from syntactic structure to semantic meaning—can enhance multimodal word grounding, with a particular focus on semantic robustness and synonym generalization. We propose a Bayesian multimodal word grounding framework that integrates linguistic features across five distinct experimental configurations. The baseline model grounds words solely through visual features and sentence descriptions. Subsequent configurations incorporate (i) word position indices, (ii) POS tags, (iii) semantic word vectors (FastText, Bojanowski et al., 2017), and (iv) large language model embeddings (BERT, Devlin et al., 2019). For semantic evaluation, we construct human-validated synonym sets spanning multiple levels of lexical divergence and evaluate the model's ability to generalize from the original training vocabulary to these synonym sets during testing.

The contributions of this work are summarized as follows:

- We introduce a unified Bayesian grounding model that accommodates diverse linguistic signals within a single generative framework.
- We design a structured set of experimental conditions to isolate the contributions of syntactic cues (indices, POS tags) and semantic representations (FastText, BERT).
- We create human-validated synonym sets with measured inter-annotator agreement to evaluate generalization beyond the training vocabulary.
- We conduct extensive experiments on the CLEVR dataset (Johnson et al., 2017), showing that semantic embeddings substantially improve generalization, with BERT offering the strongest robustness under synonym variation.

The rest of this paper is organized as follows. Section (2) reviews related work on multimodal word grounding, syntactic and semantic modeling, and synonym generalization. Section (3) introduces the problem formulation and system overview. Section (4) details the Bayesian grounding framework and inference process. Section (5) presents the experimental setup, while Section (6) reports the results and analysis. Finally, Section (7) concludes the paper and outlines directions for future research.

## 2 Related work

### 2.1 Multimodal word grounding

The challenge of grounding language in perception has long been central to cognitive science, artificial intelligence, and robotics. Harnad (1990) formalized this concept as *the symbol grounding problem*, emphasizing the need to connect abstract linguistic symbols to perceptual experiences. Building on this foundation, research in embodied cognitive science proposed that symbols emerge through sensorimotor interaction, shifting focus from symbolic manipulation to meaning derived from embodied experience. For example, Vogt (2002) defines symbols as structural couplings between perception and action but concentrates primarily on simple noun categories without addressing linguistic complexity.

Early computational systems operationalized these ideas through supervised word-to-percept mapping. Roy (2002), Roy (2005), and Roy et al. (2003) developed some of the first systems that grounded spoken words to visual referents in constrained tabletop environments. However, these models largely assumed a fixed vocabulary and did not account for deeper linguistic structures or generalizations to unseen vocabulary.

Subsequent research introduced probabilistic frameworks for multimodal language grounding. Matuszek et al. (2012) proposed a joint learning framework that integrates visual attribute classification (e.g., color and shape) with probabilistic categorial grammar-based semantic parsing to support grounded object selection. Tellex et al. (2011) introduced the Generalized Grounding Graphs (G^3^) framework, which represents language grounding as a probabilistic graphical model that links linguistic constituents to corresponding perceptual entities and robot actions. Guadarrama et al. (2013) integrated object recognition, spatial relation models, and semantic parsing to support real-time grounding and execution of complex spatial commands on robot platforms. Yu and Siskind (2013) presented a system that learns representations for multiple parts of speech—including nouns, verbs, adjectives, and prepositions—by aligning natural language sentences with video sequences, using hidden Markov models to capture temporal structure.

Aly et al. (2018), Aly and Taniguchi (2018), and Aly et al. (2017) developed an unsupervised Bayesian grounding framework that integrates 3D visual perception, spatial relation modeling, and the automatic learning of part-of-speech (POS) categories. These models combine geometric descriptors (VFH), color histograms, spatial relations, and simple syntactic cues to learn correspondences between words and perceptual entities. Although they demonstrate effective multimodal grounding, their evaluations rely on relatively small and highly controlled datasets with limited object categories, spatial relations, and colors. In contrast, our work employs synthetic 3D object scenes with fully controlled layouts, which enable precise extraction of visual features (color, centroids, and VFH descriptors) and support systematic analysis of linguistic variability. Moreover, prior models ground only the lexical forms seen during training and do not attempt to extend grounding to unseen synonyms or other forms of linguistic variation. Their reliance on syntactic signals such as POS tags, without any semantic embedding space, limits their ability to generalize beyond the observed vocabulary.

### 2.2 Semantic embeddings for word generalization

In contrast to syntactic features, semantic representations have been extensively explored in natural language processing. Embedding models such as Word2Vec (Mikolov et al., 2013), GloVe (Pennington et al., 2014), and FastText (Bojanowski et al., 2017) capture semantic similarity based on distributional patterns in large text corpora, enabling words with related meanings to occupy nearby regions in continuous vector spaces. More recently, large language models (LLMs) such as BERT (Devlin et al., 2019) provide context-sensitive embeddings that encode both syntactic and semantic dependencies. These embeddings support lexical generalization by positioning synonyms or semantically related terms close together in high-dimensional space, even when such words are not observed during training.

Despite the success of embedding models in many language-understanding tasks, their integration into multimodal word grounding remains relatively underexplored. Most existing grounding frameworks rely on symbolic or discrete lexical inputs and often assume fixed vocabularies or direct mappings between observed words and perceptual entities (Matuszek et al., 2012; Tellex et al., 2011; Guadarrama et al., 2013). While semantic embeddings have been applied in tasks such as image captioning (Bhattacharyya et al., 2024) and visual question answering (Antol et al., 2015), our preliminary work integrated static and contextual embeddings into a Bayesian multimodal grounding framework to examine synonym generalization (Shaukat et al., 2025a). However, a systematic comparison of syntactic and semantic representations within a unified Bayesian grounding framework remains limited.

Recent vision-language models such as CLIP have demonstrated strong zero-shot image—text alignment using large-scale image—caption pre-training (Radford et al., 2021). However, the present work addresses a different task: word-level Bayesian grounding, where individual words are associated with perceptual modalities and object referents using structured RGB, VFH, and centroid-based spatial features. Therefore, CLIP is considered as a related vision-language reference point rather than a directly comparable baseline.

Recent work has also explored language—vision grounding through neural-symbolic reasoning and language-conditioned control. Yi et al. (2018) proposed a neural-symbolic visual question answering framework on CLEVR that disentangles visual perception, language understanding, and symbolic program execution, showing the benefit of structured intermediate representations for visual reasoning. In robotics, Lynch and Sermanet (2021) introduced language-conditioned imitation learning over unstructured data, where natural language is used to condition visuomotor policies and improve generalization to novel instructions. These studies demonstrate the importance of combining language with visual or embodied representations. However, they differ from the present work in task formulation and modeling objective: our focus is word-level grounding in a Bayesian multimodal framework, where individual words are probabilistically associated with Color, Geometry, and Spatial Relation modalities using structured perceptual descriptors.

### 2.3 Research gap and study contributions

While prior studies have advanced multimodal grounding through visual features, syntactic structure, and supervised or unsupervised learning, they generally fail to address the critical challenge of *synonym generalization*, where multiple linguistic forms may refer to the same object but were not observed during training. Furthermore, no existing work has systematically analyzed the effect of syntactic and semantic information on word grounding performance across varying degrees of lexical divergence—that is, variation in surface word forms such as synonyms or paraphrases.

The present study advances prior work along three key dimensions. First, we introduce a multimodal Bayesian grounding framework that integrates syntactic layers (word position indices, POS tags) and semantic layers (static word vectors and contextual embeddings) within a unified probabilistic model trained using Gibbs sampling. Second, unlike prior models built on externally annotated corpora, we generate CLEVR-style 3D scenes that enable precise extraction of visual features (color histograms, centroids, and VFH descriptors) for detailed analysis of grounding performance. Third, to rigorously evaluate generalization, we design multiple human-validated synonym sets covering challenging lexical variations, allowing us to systematically analyze model robustness under synonymic shift conditions.

This integrated investigation represents a novel contribution to the multimodal word grounding literature, addressing both linguistic variability and perceptual learning within a unified Bayesian generative framework. A systematic evaluation of synonym generalization using multiple human-curated synonym sets has not been commonly addressed in prior multimodal grounding work, making this evaluation setting distinctive.

To further position the proposed framework relative to existing grounding approaches, Table 1 provides a qualitative comparison with representative prior work. A direct numerical comparison is not reported because existing grounding models are evaluated on different datasets, input modalities, supervision settings, and output tasks. In particular, the present study uses a customized CLEVR-derived 3D dataset with object-level point clouds, RGB histograms, centroid-based spatial features, and VFH descriptors, while many prior methods focus on robotic command grounding, semantic parsing, object selection, or global image–text alignment. For example, CLIP-style models are trained on image–text pairs and are typically evaluated for image-level retrieval or zero-shot recognition, whereas the present model performs object-level Bayesian word grounding across Color, Geometry, and Spatial Relation modalities. Therefore, the comparison below is methodological rather than a direct accuracy ranking.

**Table 1:** Qualitative comparison with representative prior grounding and vision-language approaches.

References	Main input	Main output/task	Difference from the proposed study
Roy (2002), Roy et al. (2003), and Roy (2005)	Spoken words and visual scenes	Learning word meanings from multimodal sensory input and visual referents	Focuses on early word–percept association in constrained settings and does not evaluate synonym generalization.
Tellex et al. (2011)	Natural-language commands and robot perception	Grounding commands to objects, places, paths, or actions	Designed for robotic command interpretation and execution rather than controlled word-level grounding across Color, Geometry, and Spatial Relation modalities.
Matuszek et al. (2012)	Language descriptions and visual object attributes	Grounded attribute learning and object-set interpretation	Uses visual attribute classifiers and probabilistic categorial grammar under a different supervision and evaluation setting.
Guadarrama et al. (2013)	Spatial language, object recognition, and robot perception	Grounding and execution of spatial commands	Focuses on human–robot spatial command grounding rather than synonym-based word grounding in controlled 3D scenes.
Aly et al. (2017), Aly and Taniguchi (2018), and Aly et al. (2018)	3D visual features and language	Bayesian multimodal grounding of spatial concepts and object categories	Closest probabilistic setting, but does not systematically evaluate semantic embeddings and synonym substitution.
CLIP-style vision-language models Radford et al., (2021)	Large-scale image–text pairs	Image–text alignment and zero-shot visual recognition	Operates at the image–text representation level and does not provide object-level Bayesian word-modality inference over RGB, centroid, and VFH features.
**Proposed framework**	3D object features, word tokens from scene descriptions, syntactic cues, and semantic embeddings	Bayesian word grounding and synonym generalization	Evaluates syntactic and semantic linguistic effects within one Bayesian framework and tests object-word synonym generalization without retraining.

The comparison is methodological because the datasets, inputs, outputs, and evaluation protocols differ across studies.

## 3 Problem formulation and system overview

### 3.1 Problem formulation

The objective is to probabilistically associate each word in a sentence with a perceptual modality and a corresponding perceptual assignment in a 3D scene. Given an input pair (*S, V*), where *S* = (ω_1_, …, ω_*N*_) denotes a sentence, and *V* represents a visual scene containing *O* segmented objects, we aim to infer the modality *m*_*i*_ and perceptual assignment *p*_*i*_ of each word ω_*i*_.

Formally, let:

- *m*_*i*_∈{Color, SpatialRelation, Geometry, Others} denotes the latent modality assigned to word ω_*i*_. The Others category covers words not assigned to the three modeled perceptual modalities, including function words and unmodeled size or material descriptors; these words are therefore not associated with a modality-specific perceptual representation in the present model.
- *p*_*i*_ denotes the perceptual assignment of word ω_*i*_. For the Color and Geometry modalities, *p*_*i*_ = *o*, where *o*∈{1, …, *O*} identifies the object whose color or geometry feature is associated with the word. For the Spatial Relation modality, *p*_*i*_ = (*o*_*r*_, *o*_*l*_), where *o*_*r*_, *o*_*l*_∈{1, …, *O*} and *o*_*r*_≠*o*_*l*_, identifies the ordered referent–landmark pair associated with the word. For Others, *p*_*i*_ = ∅.
- $\text{C}={\left\{c_{o}\right\}}_{o=1}^{O}$ denote the color features (RGB histograms);
- **S** = {*s*_*o*_*r*_, *o*_*l*__:*o*_*r*_, *o*_*l*_∈{1, …, *O*}, *o*_*r*_≠*o*_*l*_} denotes the spatial centroid–difference features for all ordered pairs of distinct objects.
- $\text{G}={\left\{g_{o}\right\}}_{o=1}^{O}$ denotes the geometric features (VFH descriptors) for each object.

The full inference problem is to estimate the joint posterior distribution over latent variables and parameters, expressed as:


$$P\left(\left\{m_{i},p_{i}\right\},\;Z,\;\theta ,\;\Phi ,\;\Pi |S,V\right)$$

where *Z* = {*z*_*c*_, *z*_*s*_, *z*_*g*_} denotes the cluster assignments for Color, Geometry, and Spatial Relation modalities, θ = {θ_*m, z*_} denotes the modality–cluster word distributions, and Φ = {μ, Σ} denotes the Gaussian parameters for continuous features, while Π denotes the case-specific modality prior (π or {π_ℓ_}) and the cluster priors {π_*c*_, π_*s*_, π_*g*_}.

This hierarchical Bayesian model jointly captures linguistic structure and perceptual evidence to ground language in a visual scene.

### 3.2 Dataset and scene generation

This study constructs a customized dataset based on CLEVR scene generator (Johnson et al., 2017), with manually authored descriptive sentences. Using Blender's Python API, we generated 240 synthetic 3D scenes, each containing 3–4 objects placed under physically plausible constraints (e.g., non-overlapping, supported, and fully visible). Scene modifications included:

- Enforcing unique object types and colors per scene.
- Controlling object orientations, sizes, and camera viewpoints.
- Adjusting lighting and brightness for visual clarity.
- Introducing logical spatial relations (e.g., on, inside, and beside) while avoiding unnatural arrangements.

The resulting dataset balances control and diversity for evaluating multimodal word grounding. Further details on the scene-generation procedure, including object-selection constraints, spatial-relation rules, sentence-construction procedure, and validation checks, are provided in Supplementary Appendix A. For each segmented object, point clouds were extracted and used to compute geometric and spatial features, such as centroids, VFH descriptors, and color histograms (see Figure 2). An example scene is shown in Figure 3.

**Figure 2:**
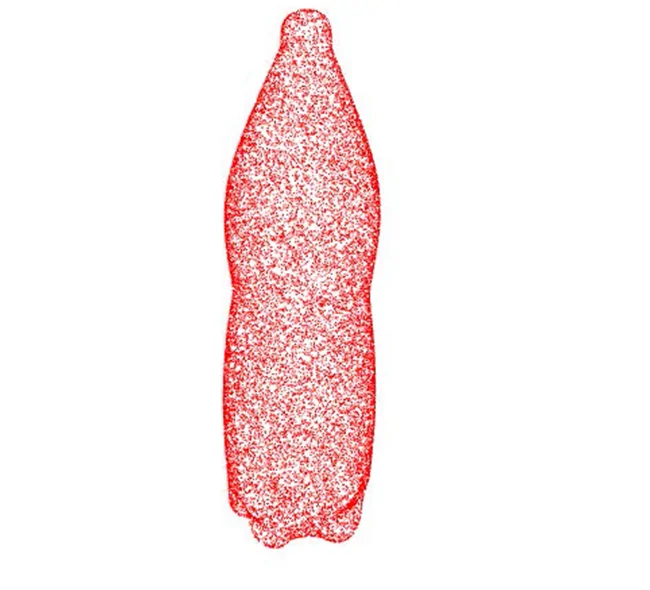
Point cloud representation of a single object (a bottle) extracted from the generated 3D scenes (adapted with permission from Shaukat et al. 2026). Point clouds serve as the basis for computing geometric descriptors (VFH), spatial centroids, and other visual features used in the multimodal grounding framework.

**Figure 3:**
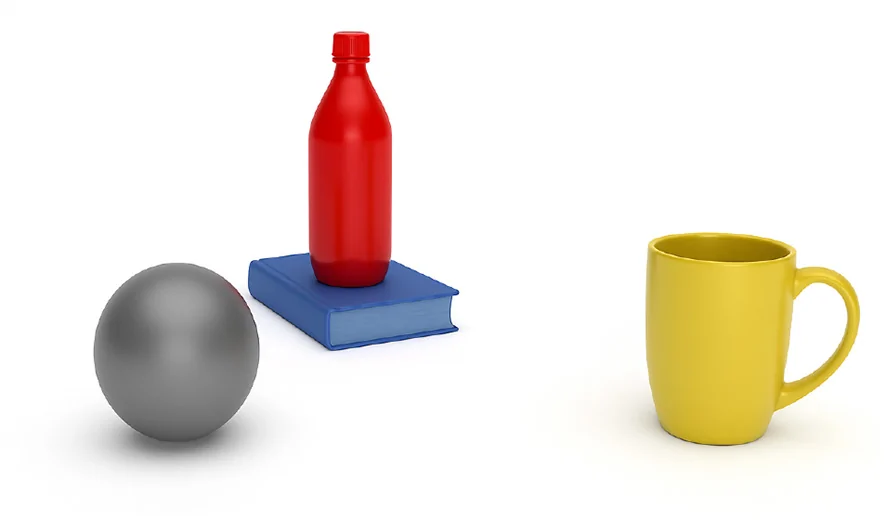
Example of a generated 3D scene used for multimodal word grounding. The scene contains distinct objects (bottle, book, cup, and ball) with varied colors, shapes, and spatial relations. The corresponding textual description is: “*The small red bottle is on the blue metallic book and the gray metallic ball is beside the big yellow cup.”*

### 3.3 Visual feature extraction

For each object segmented within a generated scene, multimodal perceptual features were extracted to support the word grounding task. Specifically, three distinct feature modalities were computed:

- **Color features:** Each object's color was represented by a 15-dimensional RGB histogram (Grauman and Leibe, 2011). The histograms capture the distributions of color intensities across the red, green, and blue channels.
- **Geometric features:** Viewpoint Feature Histograms (VFH) were computed for each object to capture detailed 3D shape descriptors, including surface normals and object geometry (Rusu et al., 2010). The resulting VFH descriptors are 308-dimensional vectors extracted directly from object-level point cloud segments.
- **Spatial features:** The 3D centroid coordinates (*x, y, z*) were calculated for each object based on its segmented point cloud, which represent the object's absolute spatial position within the scene. These centroids form the basis for modeling referent-landmark spatial relations between object pairs.

These perceptual features serve as continuous observations within the Bayesian generative grounding model.

### 3.4 Linguistic representations and experimental cases

The model incorporates enriched linguistic layers across five experimental cases:

- **Case 1 (Baseline):** The model uses the sentence words but does not incorporate any additional linguistic priors beyond the raw lexical tokens. That is, no word-position indices, no POS tags, and no semantic embeddings are used; grounding relies solely on visual features (color, spatial, and geometric) together with the observed sentence words.
- **Case 2:** Incorporation of word position indices representing the sequential position of each word within the sentence.
- **Case 3:** Addition of unsupervised POS induced through HMM to encode syntactic roles for each word.
- **Case 4:** Integration of FastText embeddings (300 dimensions) as semantic features providing continuous linguistic representations, supporting synonym generalization.
- **Case 5:** Integration of transformer based contextual embeddings (768 dimensions, bert-base-uncased) for context-sensitive semantic representation.

The vocabulary encompasses 19 object categories (e.g., *bottle, cone, book, and cylinder*), 10 colors (e.g., *red, green, and cyan*), and 8 spatial relations (e.g., *on, under, and beside*).

To rigorously evaluate generalization, multiple synonym sets were constructed specifically for object names while keeping colors and spatial relations constant. Synonym generation involved both direct and indirect substitutions, which were validated by three independent raters. Inter-rater agreement was assessed using Fleiss' kappa and reached 0.90 (Kiliç, 2015), indicating strong agreement in the validation of the synonym sets.

The complete system pipeline is illustrated in Figure 4. Each input sample consists of a manually authored text description paired with its corresponding synthetic 3D scene. We extended the CLEVR scene generator to export instance-level point clouds and object metadata. Visual features—comprising RGB color histograms, spatial centroids, and Viewpoint Feature Histograms (VFH)—are extracted from each segmented object. In parallel, linguistic representations are computed for each word using one of several syntactic or semantic encodings: word position indices, part-of-speech (POS) tags, static word vectors, or transformer-based contextual embeddings. These multimodal features are jointly processed by a hierarchical Bayesian generative model, which is trained via Gibbs sampling to infer latent modality assignments, object-word alignments, and cluster parameters across all modalities.

**Figure 4:**
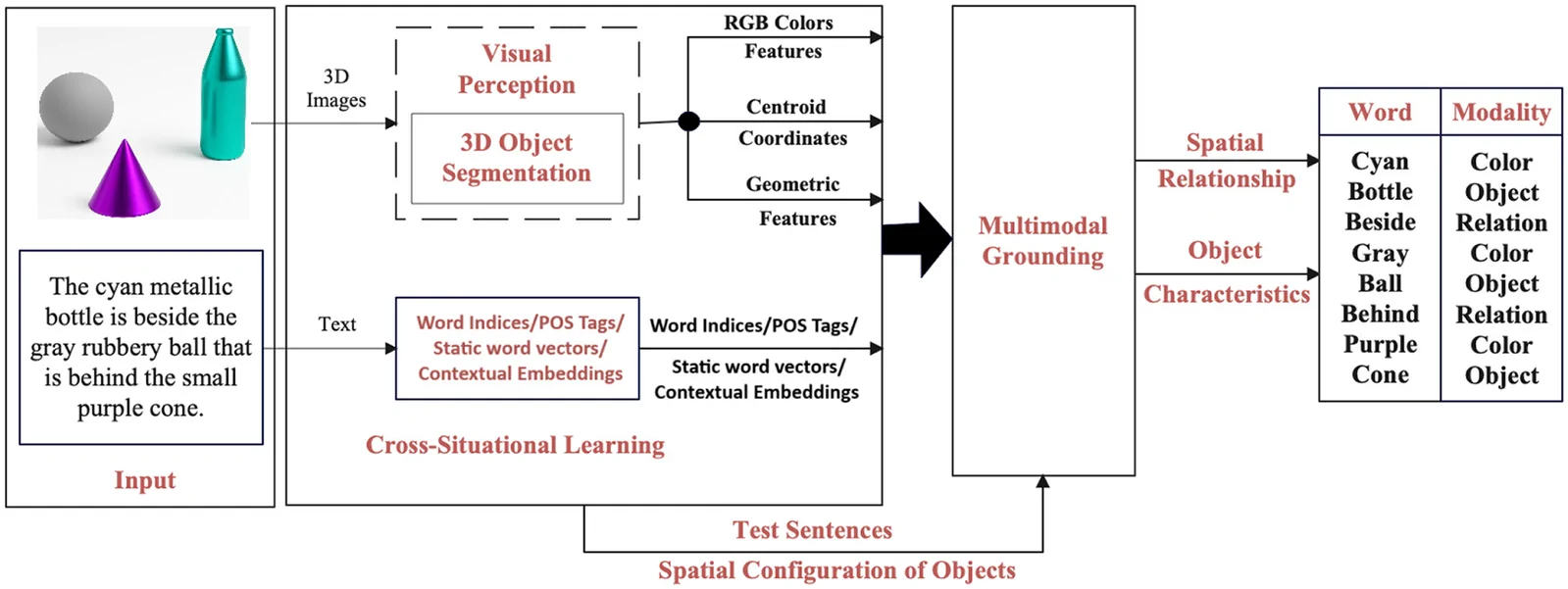
System pipeline for Bayesian multimodal word grounding (adapted with permission from Shaukat et al. 2026). Visual and linguistic features are jointly integrated through cross-situational learning under a probabilistic framework.

### 3.5 Integration of syntactic layers

The syntactic configurations incorporate word-position indices (Case 2) and unsupervised part-of-speech (POS) tags (Case 3) as linguistic features for modality inference. For each word ω_*i*_, $Z_{i}^{\text{ling}}$ denotes the observed linguistic feature, whose discrete value is denoted by ℓ_*i*_. The state ℓ_*i*_ indexes the modality prior π_ℓ_*i*__ used in the conditional inference of *m*_*i*_.

#### 3.5.1 Word-position indices (Case 2)

Each token ω_*i*_ is assigned an index corresponding to its position within the sentence. This index constitutes the observed linguistic feature $Z_{i}^{\text{ling}}$.

#### 3.5.2 Unsupervised Part-of-Speech (POS) tagging (Case 3)

For POS-based linguistic features, unsupervised POS tags are estimated before model training using a separate Gibbs sampling process, based on the approach of Stratos et al. (2016). Each sentence *w* = (*w*_1_, *w*_2_, …, *w*_*n*_) is assigned a POS tag sequence *T* = (*t*_1_, *t*_2_, …, *t*_*n*_), where the tags function as hidden states in a first-order Hidden Markov Model (HMM) (Aly and Taniguchi, 2018). The joint probability of tag sequences is expressed in Equation 1:


$$\begin{array}{l} P\left(t_{1},\ldots ,t_{n}\right)=\overset{n}{\underset{i=1}{\prod }}P\left(t_{i}|t_{i-1}\right). \end{array}$$ (1)

The emission probability models the likelihood of observing word ω_*i*_ given its tag *t*_*i*_, as shown in Equation 2:


$$\begin{array}{l} P\left(\omega_{i}|t_{i}\right). \end{array}$$ (2)

Both the transition and emission probabilities are parameterized using multinomial distributions with Dirichlet priors, as shown in Equations 3, 4:


$$\begin{array}{ll} t_{i}|t_{i-1}=t & \sim \text{Mult}\left(\phi_{t}\right),\;\phi_{t}\sim \text{Dir}\left(\alpha_{\phi }\right), \end{array}$$ (3)

$$\begin{array}{ll} \omega_{i}|t_{i}=t & \sim \text{Mult}\left(\theta_{t}\right),\;\theta_{t}\sim \text{Dir}\left(\alpha_{\theta }\right). \end{array}$$ (4)

Given an unannotated training corpus *W* = {*w*_1_, …, *w*_*m*_}, the most probable tag assignments *T* are inferred by maximizing the objective in Equation 5:


$$\begin{array}{l} P\left(T,W\right)=\underset{\left(T,w\right)\in \left(T,W\right)}{\prod }\left(\overset{n}{\underset{i=1}{\prod }}P\left(t_{i}|t_{i-1},\phi_{t}\right)P\left(\omega_{i}|t_{i},\theta_{t}\right)\right). \end{array}$$ (5)

The latent POS tags are estimated using Gibbs sampling from the posterior distribution in Equation 6:


$$\begin{array}{l} P\left(T_{i},T^{\left(i\right)}|T_{-i},W,T^{\left(-i\right)},w,\alpha_{\phi },\alpha_{\theta }\right). \end{array}$$ (6)

Once learned, the resulting POS tag *t*_*i*_ for each word ω_*i*_ is used as the observed linguistic feature $Z_{i}^{\text{ling}}=t_{i}$. The corresponding discrete state is denoted by ℓ_*i*_ = *t*_*i*_, which indexes the modality prior π_ℓ_*i*__. This integration enables the model to leverage both syntactic word properties and cross-situational visual co-occurrence patterns during grounding.

### 3.6 Integration of semantic embeddings

In addition to syntactic layers, the model further incorporates semantic embeddings as an additional linguistic layer to better capture word semantics and lexical variability. This allows the framework to generalize over synonyms and fine-grained lexical variations that may not be captured by discrete syntactic tags alone. Two types of semantic embeddings were integrated into the model depending on the experimental case:

#### 3.6.1 Static word vectors (Case 4)

In Case 4, each word ω_*i*_ is mapped to a continuous embedding vector ${\text{e}}_{i}\in {\mathbb{R}}^{300}$ using pretrained FastText word vectors. These embeddings capture subword-level semantic regularities and provide smooth representations for words, including those not seen during training. The embedding **e**_*i*_ serves as the observed linguistic feature $Z_{i}^{\text{ling}}$, replacing POS tags used in Case 3, as defined in Equation 7:


$$\begin{array}{ll} Z_{i}^{\text{ling}} & =e_{i}. \end{array}$$ (7)

Given this input, modality assignments in the semantic-embedding case are sampled from a single global modality prior:


$$\begin{array}{l} \pi \sim \text{Dir}\left(\lambda \right), \end{array}$$ (8)

$$\begin{array}{l} m_{i}\sim \text{Cat}\left(\pi \right). \end{array}$$ (9)

Since *e*_*i*_ is continuous, it does not index a category-specific modality prior. Modality assignments therefore use the global prior in Equations 8, 9, while the embedding contributes to inference through the following embedding-aware lexical score, which replaces the exact-token lexical factor in the conditional sampling equations for Cases 4–5:


$$\begin{array}{lll} {\tilde{\theta }}_{m,z}(\omega_{i}|e_{i}) & = & \left(1-\rho )\theta_{m,z}(\omega_{i}\right) \end{array}\begin{array}{ll} + & \rho \underset{v\in V_{\text{train}}}{\sum^{\text{​}}}\theta_{m,z}(v)\frac{\exp(\cos(e_{i},e_{v})/\tau )}{\underset{u\in V_{\text{train}}}{\sum^{\text{​}}}\exp(\cos(e_{i},e_{u})/\tau )}. \end{array}$$ (10)

*V*_train_ denotes the vocabulary observed during training, ρ∈[0, 1] is a fixed semantic mixing coefficient, and τ>0 is a fixed softmax temperature. The vector *e*_*i*_ represents the current word occurrence ω_*i*_, while *e*_*v*_ represents a word *v*∈*V*_train_.

For FastText, *e*_*i*_ and *e*_*v*_ are fixed, context-independent pretrained word vectors. For BERT, a word does not have a single context-independent representation because its embedding depends on the sentence in which it occurs. We therefore distinguish between the contextual representation *e*_*i*_ of the current word occurrence and the training-corpus contextual prototype *e*_*v*_ of vocabulary word *v*.

For a synonym word ω_*i*_∉*V*_train_, the exact-token component is set to θ_*m, z*_(ω_*i*_) = 0. Its embedding-aware lexical score is therefore determined entirely through similarity-weighted transfer from words in *V*_train_ using Equation 10. The synonym word is not introduced as a new learned entry in θ_*m, z*_.

#### 3.6.2 Transformer-based LLM embeddings (Case 5)

In Case 5, contextual semantic embeddings are extracted using the bert-base-uncased transformer model. Each token ω_*i*_ is mapped to a 768-dimensional contextual vector:


$$Z_{i}^{\text{ling}}=e_{i}\in {\mathbb{R}}^{768}.$$

Let $I_{v}=\left\{j:\omega_{j}=v\right\}$ denote the set of occurrences of the word *v* in the training descriptions, and let $f_{\text{BERT}}\left(\omega_{j},s_{j}\right)\in {\mathbb{R}}^{768}$ denote the contextual representation of occurrence ω_*j*_ in the sentence *s*_*j*_. The training-corpus prototype is defined as:


$${ē}_{v}=\frac{1}{\left|I_{v}\right|}\underset{j\in I_{v}}{\sum }f_{\text{BERT}}\left(\omega_{j},s_{j}\right),\;e_{v}=\frac{{ē}_{v}}{||{ē}_{v}|{|}_{2}}.$$ (10a)

For the current occurrence ω_*i*_ in sentence *s*_*i*_,


$$e_{i}=\frac{f_{\text{BERT}}\left(\omega_{i},s_{i}\right)}{||f_{\text{BERT}}\left(\omega_{i},s_{i}\right)|{|}_{2}}.$$ (10b)

For an unseen synonym, *e*_*i*_ is computed from its current test-sentence context without using its grounding label or perceptual category. The prototypes *e*_*v*_ are constructed from the training descriptions and remain fixed during grounding inference. In the BERT configuration, Equation 10 compares the contextual representation *e*_*i*_ of the current word occurrence with the prototype *e*_*v*_ of each word *v*∈*V*_train_.

## 4 Bayesian multimodal grounding framework

### 4.1 Generative process

The proposed Bayesian model probabilistically aligns linguistic tokens with multimodal perceptual features extracted from 3D scenes. The model is a hierarchical generative framework in which words and color, spatial, and geometric observations are modeled as draws conditioned on latent variables and hierarchical priors. The generative process is as follows:

#### 4.1.1 Linguistic generation

For each word ω_*i*_ in the sentence, the model may incorporate an additional linguistic feature $Z_{i}^{\text{ling}}$, depending on the experimental case. This feature is observed in Cases 2–5, but it *conditions the modality prior only in the discrete linguistic settings* (Cases 2–3). In the baseline configuration (Case 1), no linguistic feature is used and $Z_{i}^{\text{ling}}$ is absent; in the semantic-embedding settings (Cases 4–5), $Z_{i}^{\text{ling}}$ corresponds to a continuous vector and therefore does not introduce a discrete conditioning index for π_ℓ_.

- Case 1: no linguistic feature is used.
- Case 2: $Z_{i}^{\text{ling}}$ = word position index,
- Case 3: $Z_{i}^{\text{ling}}$ = POS tag,
- Cases 4–5: $Z_{i}^{\text{ling}}$ = semantic embedding (FastText or BERT),

In Cases 2–3, $Z_{i}^{\text{ling}}$ is an observed discrete linguistic feature whose value is denoted by ℓ_*i*_. A modality prior π_ℓ_ is drawn for each possible discrete linguistic state ℓ is defined in Equation 11.


$$\begin{array}{l} \pi_{\ell }\sim \text{Dir}\left(\lambda \right),\;\ell =1,\ldots ,K. \end{array}$$ (11)

The value of *K* depends on the syntactic configuration: it is the number of word-position states in Case 2 and the number of induced POS-tag states in Case 3. For each word ω_*i*_, the observed state ℓ_*i*_ determines the modality prior π_ℓ_*i*__ used for its modality assignment. In Cases 4–5, $Z_{i}^{\text{ling}}$ is a continuous FastText or BERT embedding; therefore, no discrete linguistic state ℓ_*i*_ or corresponding modality prior π_ℓ_*i*__ is defined. Modality assignments use the global modality prior π, while the semantic embeddings are incorporated into the conditional sampling equations using the embedding-aware lexical score in Equation 10.

The modality assignment for each word is sampled from a categorical distribution that depends on the experimental case:


$$m_{i}\sim \{\begin{array}{ll} Cat(\pi ), & for\;Case\;1,\\ Cat(\pi_{\ell_{i}}), & for\;Cases\;2–3,\\ Cat(\pi ), & for\;Cases\;4–5. \end{array}$$

The perceptual assignment for each word is sampled from a categorical prior:


$$\begin{array}{l} p_{i}\sim \text{Cat}\left(\delta \right). \end{array}$$

The support of *p*_*i*_ contains object indices for the Color and Geometry modalities, ordered pairs of distinct object indices (*o*_*r*_, *o*_*l*_), where *o*_*r*_≠*o*_*l*_, for the Spatial Relation modality, and the null assignment ∅ for the Others category.

The observed word token is generated from the modality–cluster lexical distribution: ω_*i*_~Cat(θ_*m*_*i*_, *z*_), with θ_*m, z*_~Dir(γ). In Cases 4–5, the embedding-aware lexical score in Equation 10 is used instead of θ_*m, z*_(ω_*i*_) when computing the conditional sampling probabilities.

#### 4.1.2 Visual feature generation

For each observed object *o* in the scene, its multimodal visual features are generated as follows:

- **Color modality:** The color cluster assignment for object *o* is sampled as:
$$z_{o}^{c}\sim \text{Cat}\left(\pi_{c}\right),\;\pi_{c}\sim \text{Dir}\left(\alpha_{c}\right),$$
  followed by the color feature,
$$c_{o}\sim N\left(\mu_{z_{o}^{c}}^{c},\Sigma_{z_{o}^{c}}^{c}\right).$$
  Each cluster's Gaussian parameters are drawn independently from a Normal–Inverse–Wishart prior,
$$\mu_{k}^{c},\;\Sigma_{k}^{c}\sim \text{NIW}\left(\beta_{c}\right),\;k=1,\ldots ,K_{c}.$$
- **Spatial relation modality:** The spatial cluster assignment for object pair *q* = (*o*_*r*_, *o*_*l*_) where *o*_*r*_ and *o*_*l*_ denote the referent and landmark objects, respectively, is sampled as
$$z_{q}^{s}\sim \text{Cat}\left(\pi_{s}\right),\;\pi_{s}\sim \text{Dir}\left(\alpha_{s}\right),$$
  and the spatial feature is drawn from the corresponding Gaussian component:
$$s_{q}\sim N\left(\mu_{z_{q}^{s}}^{s},\Sigma_{z_{q}^{s}}^{s}\right).$$
  Each spatial cluster has its own Gaussian parameters sampled from a Normal–Inverse–Wishart prior:
$$\mu_{k}^{s},\;\Sigma_{k}^{s}\sim \text{NIW}\left(\beta_{s}\right),\;k=1,\ldots ,K_{s}.$$
- **Geometry modality:** The geometric cluster assignment for object *o* is drawn as:
$$z_{o}^{g}\sim \text{Cat}\left(\pi_{g}\right),\;\pi_{g}\sim \text{Dir}\left(\alpha_{g}\right),$$
  and the geometric feature is generated from the corresponding Gaussian component:
$$g_{o}\sim N\left(\mu_{z_{o}^{g}}^{g},\Sigma_{z_{o}^{g}}^{g}\right).$$
  Each geometric cluster is parameterized by its own Gaussian distribution with NIW prior:
$$\mu_{k}^{g},\;\Sigma_{k}^{g}\sim \text{NIW}\left(\beta_{g}\right),\;k=1,\ldots ,K_{g}.$$

Figure 5 reflects the formulation described above. Cases 2–3 use the modality prior π_ℓ_*i*__, whereas Case 1 and Cases 4–5 use the global modality prior π. In Cases 4–5, the embedding-aware lexical score in Equation 10 is used when computing the conditional sampling probabilities. $Z_{o}^{s}$ and *s*_*o*_ denote the pair-indexed variables $Z_{q}^{s}$ and *s*_*q*_, where *q* = (*o*_*r*_, *o*_*l*_); the nested *O* and *O*−1 plates represent all ordered pairs of distinct objects. $\phi_{k}^{m}$ denotes the Gaussian parameters $\left(\mu_{k}^{m},\Sigma_{k}^{m}\right)$ for cluster *k* of modality *m*∈{*c, s, g*}. Table 2 summarizes the definitions of all model variables and hyperparameters used in the generative process.^1^ The hyperparameter values used in the experiments, together with their justification, are reported in Supplementary Appendix A.

**Figure 5:**
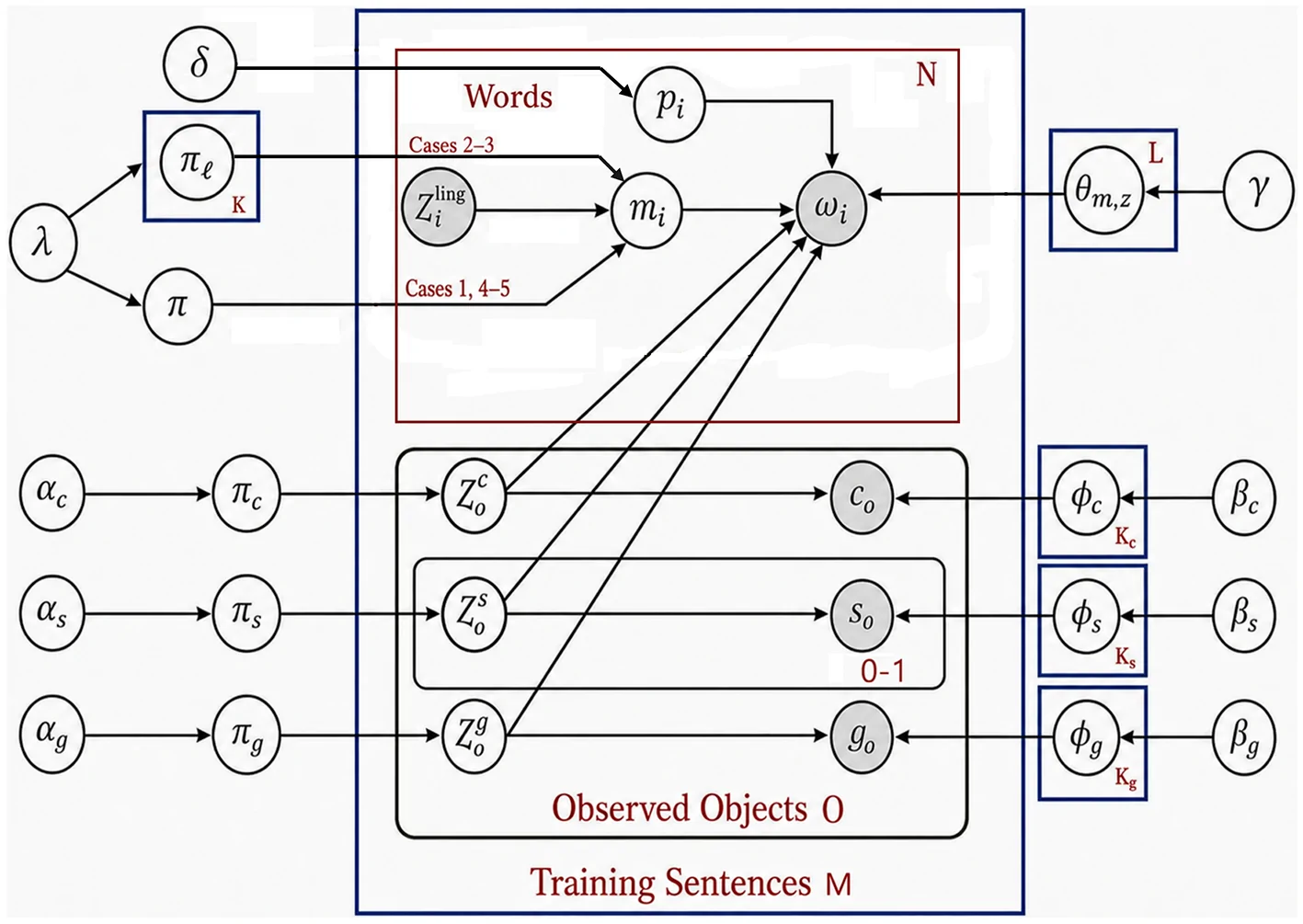
Graphical representation of the Bayesian generative model (adapted with permission from Shaukat et al. 2026).

**Table 2:** Definitions of the generative model parameters in the different modalities.

**Parameter**	Definition
δ	Hyperparameter of the categorical prior over the perceptual-assignment variable *p*_*i*_.
*p*_*i*_	Perceptual-assignment variable associated with word ω_*i*_: an object index *o* for Color and Geometry, an ordered pair *q* = (*o*_*r*_, *o*_*l*_) of distinct object indices for Spatial Relation, and ∅ for Others.
λ	Hyperparameter of the modality prior distributions π_ℓ_ for Cases 2–3 and global π for Case 1 and Cases 4–5
*m*_*i*_	Index of word modality, where *m*_*i*_∈{Color, Geometry, SpatialRelation, Others}. The *Others* category includes words that are not assigned to the Color, Geometry, or Spatial Relation modalities, such as function words (“the,” “and”) and size or material terms (“big,” “metallic”).
ℓ_*i*_	Discrete linguistic state used to index the modality prior π_ℓ_*i*__ in Cases 2–3
π_ℓ_	Modality prior indexed by the discrete linguistic state ℓ in Cases 2–3
π	Global modality prior used in Case 1 and Cases 4–5
γ	Hyperparameter of the distribution θ_*m, z*_
*L*	Number of word-distribution categories, where *L* = *K*_*c*_+*K*_*s*_+*K*_*g*_+1; the additional +1 corresponds to the Others category.
θ_*m, z*_	Word distribution over modality–cluster pairs
${\tilde{\theta }}_{m,z}\left(\omega_{i}|e_{i}\right)$	Embedding-aware lexical score used in Cases 4–5
*e*_*i*_	Semantic embedding of the current word occurrence ω_*i*_ in Cases 4–5
*e*_*v*_	Embedding representation associated with a training-vocabulary word *v*∈*V*_train_
*V*_train_	Vocabulary observed during training
ρ	Fixed semantic mixing coefficient used in the embedding-aware lexical score
τ	Fixed softmax temperature used for embedding-similarity weighting
α_*c*_	Hyperparameter of the distribution π_*c*_
β_*c*_	Hyperparameter of the distribution ϕ_*c*_
*K*_*c*_	Number of color clusters
α_*s*_	Hyperparameter of the distribution π_*s*_
β_*s*_	Hyperparameter of the distribution ϕ_*s*_
*K*_*s*_	Number of Spatial Relation clusters
α_*g*_	Hyperparameter of the distribution π_*g*_
β_*g*_	Hyperparameter of the distribution ϕ_*g*_
*K*_*g*_	Number of object/geometry clusters
$Z_{o}^{c}$	Index of object color clusters
$Z_{q}^{s}$	Index of Spatial Relation clusters for ordered object pair *q* = (*o*_*r*_, *o*_*l*_)
$Z_{o}^{g}$	Index of object/geometry clusters

### 4.2 Inference via Gibbs sampling

Inference in the proposed Bayesian multimodal grounding framework is performed using Gibbs sampling, where latent variables are iteratively sampled from their conditional posterior distributions. Each sampling step is detailed below:

To express the cluster-sampling updates consistently across all experimental cases, we define a case-dependent lexical contribution:


$$\psi_{m,k}^{\left(i\right)}=\{\begin{array}{ll} \theta_{m,k}(\omega_{i}), & for\;Cases\;1–3,\\ {\tilde{\theta }}_{m,k}(\omega_{i}|e_{i}), & for\;Cases\;4–5, \end{array}$$

$\psi_{m,k}^{\left(i\right)}$ denotes the lexical contribution of word ω_*i*_ under modality *m* and cluster *k*. For Cases 1–3, it corresponds to the standard word probability θ_*m, k*_(ω_*i*_). For Cases 4–5, it corresponds to the embedding-aware lexical score defined in Equation 10.

#### 4.2.1 Color cluster sampling (sampling $Z_{o}^{c}$)

For each object, its color cluster assignment is sampled conditioned on its observed color feature *c*_*o*_ and word tokens aligned with the Color modality. The Color cluster assignment is sampled according to Equation 12:


$$\begin{array}{l} P\left(Z_{o}^{c}=k|\cdot \right)\propto \pi_{c}\left(k\right)N\left(c_{o}|\mu_{c,k},\Sigma_{c,k}\right)\underset{i:m_{i}=\text{Color},p_{i}=o}{\prod }\psi_{\text{Color},k}^{\left(i\right)}. \end{array}$$ (12)

#### 4.2.2 Spatial relation cluster sampling (sampling $Z_{q}^{s}$)

For each ordered object pair *q* = (*o*_*r*_, *o*_*l*_), where *o*_*r*_ and *o*_*l*_ denote the referent and landmark objects, respectively, the Spatial Relation cluster assignment is sampled based on the spatial feature *s*_*q*_ (the centroid difference between the referent and the landmark) and related words. The Spatial Relation cluster assignment is sampled according to Equation 13:


$$\begin{array}{lll} P(Z_{q}^{s}=k|\cdot ) & \propto & \pi_{s}(k)N(s_{q}|\mu_{s,k},\Sigma_{s,k})\underset{i:m_{i}=\text{SpatialRelation},p_{i}=q}{\prod^{\text{​}}} \end{array}\begin{array}{l} \psi_{\text{SpatialRelation},k}^{\left(i\right)}. \end{array}$$ (13)

#### 4.2.3 Geometry cluster sampling (sampling $Z_{o}^{g}$)

For each object, its Geometry cluster assignment is sampled using the VFH descriptor *g*_*o*_ and assigned words. The Geometry cluster assignment is sampled according to Equation 14:


$$\begin{array}{lll} P(Z_{o}^{g}=k|\cdot ) & \propto & \pi_{g}(k)N(g_{o}|\mu_{g,k},\Sigma_{g,k})\underset{i:m_{i}=\text{Geometry},\;p_{i}=o}{\prod^{\text{​}}} \end{array}\begin{array}{l} \psi_{\text{Geometry},k}^{\left(i\right)}. \end{array}$$ (14)

Therefore, in Cases 4–5, semantic embeddings contribute to modality assignment via ${\tilde{\theta }}_{m,z}\left(\omega_{i}|e_{i}\right)$ and to the conditional updates of the Color, Geometry, and Spatial Relation clusters through the shared case-dependent lexical contribution $\psi_{m,k}^{\left(i\right)}$.

#### 4.2.4 Modality assignment sampling (sampling *m*_*i*_)

The modality assignment for each word is sampled according to the experimental case. The modality assignment is sampled according to the experimental configuration in Equation 15:


$$P(m_{i}=m|\cdot )\propto \{\begin{array}{ll} \pi (m)\theta_{m,z}(\omega_{i}), & \text{for Case 1,}\\ \pi_{\ell_{i}}(m)\theta_{m,z}(\omega_{i}), & \text{for Cases 2}–\text{3,}\\ \pi (m){\tilde{\theta }}_{m,z}(\omega_{i}|e_{i}), & \text{for Cases 4}–\text{5,} \end{array}$$ (15)

where π_ℓ_*i*__(*m*) denotes the probability of modality *m* under the modality prior indexed by the discrete linguistic state ℓ_*i*_ in Cases 2–3, and *z* denotes the cluster associated with perceptual assignment *p*_*i*_ under candidate modality *m*. In Case 1 and Cases 4–5, no discrete linguistic state indexes the modality prior, so the global prior π(*m*) is used. In Cases 4–5, the semantic embedding enters the conditional modality update through the embedding-aware lexical score ${\tilde{\theta }}_{m,z}\left(\omega_{i}|e_{i}\right)$.

#### 4.2.5 Perceptual-assignment sampling (sampling *p*_*i*_)

The same case-dependent lexical contribution $\psi_{m,k}^{\left(i\right)}$ is used when sampling the perceptual assignment *p*_*i*_ associated with the word ω_*i*_. For the Color and Geometry modalities, the candidate set comprises all objects in the scene, and *r* = *o*, with *o*∈{1, …, *O*}, denotes one candidate object. For the Spatial Relation modality, the candidate set comprises all eligible ordered object pairs, and *r* = *q* = (*o*_*r*_, *o*_*l*_), with *o*_*r*_, *o*_*l*_∈{1, …, *O*} and *o*_*r*_≠*o*_*l*_, denotes one candidate referent–landmark pair. For the Others category, *p*_*i*_ = ∅, and no perceptual likelihood is evaluated. Thus, for the Color, Geometry, and Spatial Relation modalities, the sampler evaluates all candidate objects or ordered object pairs according to the assigned modality. The perceptual assignment *p*_*i*_ is sampled according to Equation 16:


$$\begin{array}{l} P\left(p_{i}=r|\cdot \right)\propto \psi_{m_{i},z_{r}}^{\left(i\right)}N\left(x_{r}^{\left(m_{i}\right)}|\mu_{m_{i},z_{r}},\Sigma_{m_{i},z_{r}}\right), \end{array}$$ (16)

where $x_{r}^{\left(m_{i}\right)}$ denotes the corresponding observed feature (*c*_*o*_, *s*_*q*_, or *g*_*o*_) under modality *m*_*i*_, and *z*_*r*_ denotes the cluster assignment associated with candidate *r*.

#### 4.2.6 Word distribution sampling (sampling θ_*m, z*_)

Each modality-cluster word distribution is sampled from its conjugate Dirichlet posterior. The modality-cluster word distribution is sampled according to Equation 17:


$$\begin{array}{l} \theta_{m,z}\sim \text{Dir}\left(\gamma +\text{word counts assigned to}\left(m,z\right)\right). \end{array}$$ (17)

The embedding-aware lexical score ${\tilde{\theta }}_{m,z}\left(\omega_{i}|e_{i}\right)$ is not sampled as an additional Dirichlet-distributed parameter. Instead, it is computed from the sampled modality–cluster word distribution θ_*m, z*_ and the semantic similarities between the current embedding *e*_*i*_ and the embedding representations of words in *V*_train_, as defined in Equation 10.

#### 4.2.7 Modality prior sampling

For Cases 2–3, the modality prior is indexed by the discrete linguistic state ℓ. The discrete-state modality prior is updated according to Equation 18. The prior for each discrete state is updated as:


$$\begin{array}{l} \pi_{\ell }\sim \text{Dir}\left(\lambda +\text{modality counts for}\ell \right). \end{array}$$ (18)

For Case 1 and Cases 4–5, no discrete linguistic state is used to index the modality prior. Therefore, a single global modality prior is updated according to Equation 19.


$$\begin{array}{l} \pi \sim \text{Dir}\left(\lambda +\text{global modality counts}\right). \end{array}$$ (19)

#### 4.2.8 Cluster prior sampling (sampling π_*c*_, π_*s*_, π_*g*_)

The cluster priors are sampled according to Equation 20.


$$\begin{array}{cc} \pi_{c} & \sim \text{Dir}(\alpha_{c}+\text{counts of}\;Z_{o}^{c}),\\ \pi_{s} & \sim \text{Dir}(\alpha_{s}+\text{counts of}\;Z_{q}^{s}),\\ \pi_{g} & \sim \text{Dir}(\alpha_{g}+\text{counts of}\;Z_{o}^{g}). \end{array}$$ (20)

#### 4.2.9 Gaussian parameter sampling (sampling μ, Σ)

For each modality and cluster, Gaussian parameters are sampled from Normal-Inverse-Wishart posteriors according to Equation 21.


$$\begin{array}{cc} \left(\mu_{c,k},\Sigma_{c,k}\right) & \sim \text{NIW posterior based on}\{c_{o}:Z_{o}^{c}=k\},\\ \left(\mu_{s,k},\Sigma_{s,k}\right) & \sim \text{NIW posterior based on}\{s_{q}:Z_{q}^{s}=k\},\\ \left(\mu_{g,k},\Sigma_{g,k}\right) & \sim \text{NIW posterior based on}\{g_{o}:Z_{o}^{g}=k\}. \end{array}$$ (21)

The entire Gibbs sampling process is iteratively repeated until convergence. The full inference procedure is summarized in Algorithm 1.

Algorithm 1 Gibbs sampling for Bayesian multimodal word grounding. 
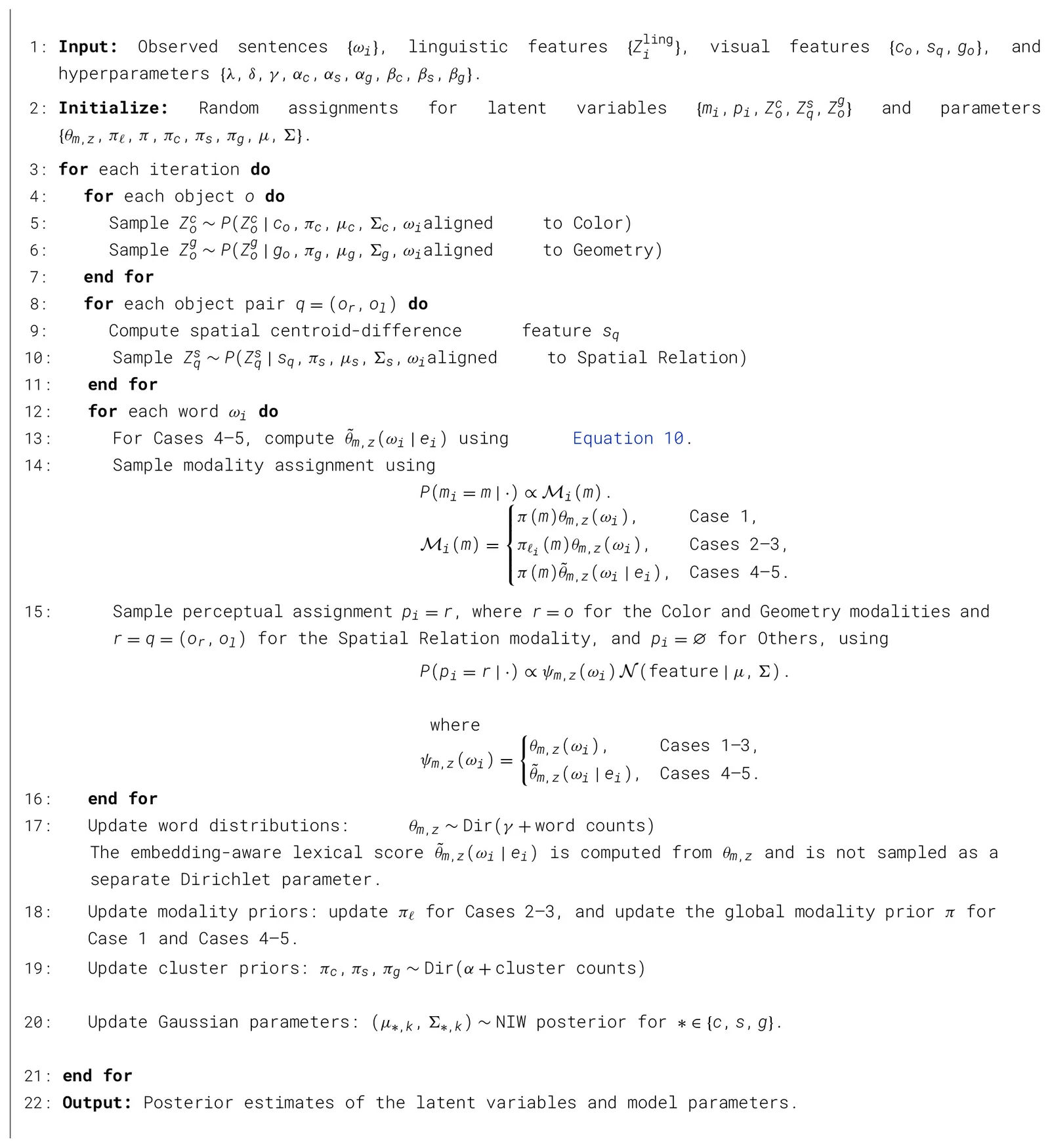

## 5 Experiments and evaluation

### 5.1 Experimental setup

As detailed in Section 3.2, we use a dataset of 240 synthetic 3D scenes, each paired with a human-authored sentence describing object attributes and spatial configurations. The data is split into 70% training and 30% testing. Each scene contains 3–4 segmented objects with extracted visual features from the Color, Geometry, and Spatial Relation modalities. The model is trained on the original descriptions and evaluated on both the original and synonym-substituted test sets.

Although the dataset contains 240 scenes, the model does not learn directly from raw images. Each scene is first represented through extracted perceptual descriptors, including RGB color histograms, VFH geometric descriptors, and centroid-based spatial features. The Bayesian model, therefore, learns word-grounding associations over these continuous descriptors rather than learning visual representations from pixels. This controlled setup is sufficient for the objective of isolating the effect of different linguistic representations under the same perceptual feature space, although it does not claim unrestricted generalization to real-world scenes.

To assess synonym generalization, we define three controlled synonym sets (S1, S2, and S3), involving substitutions only for object category words. These substitutions are introduced only during testing in Case 4 and Case 5, and none of the synonym words appear in the training data. This ensures a rigorous evaluation of the model's ability to generalize to out-of-vocabulary lexical variants.

“Out-of-vocabulary” refers to words that are not observed in the training descriptions of the proposed grounding model. It does not imply that these words are unknown to the pre-trained embedding models. In Cases 4 and 5, synonym substitutions are represented through FastText or BERT embeddings and enter the model through the embedding-aware lexical score ${\tilde{\theta }}_{m,z}\left(\omega_{i}|e_{i}\right)$. Therefore, this evaluation measures lexical transfer through a pre-trained semantic space within the Bayesian grounding framework, rather than open-ended discovery of entirely new object concepts.

Each experiment is repeated five times per case, using different random seeds. A summary of the five experimental configurations is provided in Table 3. Final results are reported as the mean grounding accuracy, together with color, spatial relation, and object-specific accuracies.

**Table 3:** Overview of experimental cases.

Feature	Case 1	Case 2	Case 3	Case 4	Case 5
Input data	Text + visual features	Case 1 + word index	Case 1 + POS tags	Case 1 + FastText	Case 1 + BERT
Unseen word grounding	No	No	No	Yes	Yes
Synonym test sets used	No	No	No	Yes	Yes
Expected impact	Baseline learning model	Impact of word-position awareness	Impact of syntactic information	Leverage semantics	Leverage deeper semantics

### 5.2 Synonym set construction

To fulfill one of the objectives of this research—evaluating how well a multimodal grounding model can generalize to unseen synonyms—we constructed multiple human-validated synonym sets targeting object-word replacements. These sets were created to evaluate how well the model can match different word expressions to the correct visual objects.

Each synonym set consists of alternative expressions for 19 original object words (e.g., *cup, book*, and *car*) while keeping all other vocabulary—including color terms, spatial relations, and functional words—unchanged. This ensures that generalization performance reflects the effect of object-word substitutions only.

Synonym substitution was restricted to object-category words because the synonym sets were designed to evaluate lexical variation in object grounding. Object names naturally allow meaningful synonym or near-synonym substitutions, such as “car”–“automobile”, “cup”–“mug”, and “book”–“novel”. These substitutions directly test whether semantic embeddings can link unseen object labels to the same Geometry modality.

Color and spatial relation terms were kept unchanged because they do not provide the same type of synonym structure in this controlled dataset. Basic color words such as “red,” “blue,” and “yellow” are visually defined category labels; replacing them with alternative expressions would often change the color category or introduce ambiguity rather than create a true synonym. Similarly, spatial relation words such as “on,” “under,” “inside,” and “beside” are tied to centroid-based geometric configurations. Replacing them with paraphrases would require a different spatial relation representation and evaluation protocol. Therefore, keeping colors and spatial relations fixed allows the synonym experiment to isolate lexical generalization for object words while preserving the same visual content and spatial arrangement of the scene.

#### 5.2.1 Synonym set design

Three synonym sets were created, each varying in semantic closeness to the original terms:

- **Synonyms 1:** Direct lexical equivalents (e.g., *cup*→*mug, car*→*automobile*).
- **Synonyms 2:** Less direct, but still semantically plausible (e.g., *cup*→*teacup, car*→*vehicle*).
- **Synonyms 3:** Indirect associations (e.g., *cup*→*coffee, car*→*Audi*), requiring deeper semantic reasoning.

Each set was annotated and validated by three independent human annotators. Final selections were made based on majority agreement and semantic plausibility. The inter-rater agreement was computed using Fleiss' kappa, achieving a high score of 0.90, indicating substantial annotation consistency. The annotator instructions, synonym-selection criteria, validation procedure, and representative examples for S1–S3 are provided in Supplementary Appendix A.

#### 5.2.2 Embedding-based similarity evaluation

To assess the semantic proximity between original object labels and their synonymic replacements, we computed cosine similarity scores using both FastText and BERT representations. For FastText, cosine similarity was computed between static pre-trained word vectors. For BERT, because token embeddings are context-dependent, cosine similarity was computed using the same contextual embedding representation adopted in the semantic-embedding configuration, so that the similarity analysis remains consistent with the embedding-aware lexical score in Equation 10. Due to space constraints, Table 4 presents a representative subset of object–synonym pairs sampled from the three synonym sets used in our generalization experiments.

**Table 4:** Cosine similarity scores for representative object–synonym pairs sampled from Synonym Sets 1–3.

Object–Synonym pair	FastText	LLM (BERT)
Synonyms 1		
car–automobile	0.78	0.92
glass–stemware	0.61	0.91
canister–can	0.31	0.89
crate–box	0.59	0.97
book–novel	0.67	0.95
Synonyms 2		
car–vehicle	0.79	0.90
glass–wineglass	0.74	0.83
canister–tin	0.44	0.79
crate–chest	0.47	0.89
book–textbook	0.69	0.86
Synonyms 3		
car–Audi	0.51	0.73
glass–soda-lime	0.56	0.67
canister–Coke	0.27	0.63
crate–goods	0.43	0.69
book–Bible	0.53	0.69

Across all sets, transformer-based embeddings consistently yield higher similarity scores than static embeddings, particularly for Synonym Sets 1 and 2, which were constructed with strong inter-annotator agreement based on human validation. For example, in Synonym Set 1, direct substitutions such as *car-automobile* and *glass-stemware* achieve scores of 0.92 and 0.91 in the contextual embedding space, compared to 0.78 and 0.61 in FastText. In Synonym Set 3—comprising less conventional or contextually nuanced pairs such as *canister-Coke*—the gap widens further: BERT captures moderate similarity (0.63) while FastText assigns a much lower value (0.27). These findings align with human semantic intuitions, confirming that LLM embeddings are more consistent with perceived synonymy.

Overall, FastText exhibits greater variance and limited sensitivity to low-frequency or compound expressions, underscoring its weaker generalization capacity. In contrast, Transformer based embeddings leverage contextual semantics to preserve alignment even for abstract or culturally contingent terms. These results reinforce the conclusion that robust synonym generalization—especially under linguistic variability—relies not only on lexical substitution but also on the representational depth of the embedding space. The semantic alignment patterns observed align closely with human judgments, lending further credibility to the model's behavior in the synonym generalization experiments.

### 5.3 Evaluation metrics

To assess the performance of our multimodal word grounding model, we compute both overall and modality-specific grounding accuracies.

- **Overall grounding accuracy:** The percentage of correctly grounded words across all modalities (Color, Geometry, and Spatial Relation).
- **Color accuracy:** The accuracy of grounding words that refer to color attributes, based on correct alignment to the target object with matching color features.
- **Spatial Relation accuracy:** The accuracy of grounding spatial relation terms (e.g., “on,” “beside”) to correct referent-landmark object pairs.
- **Object accuracy:** The accuracy of grounding words referring to object shape or identity (e.g., “cup,” “ball”) based on VFH geometric features.

These metrics were selected for their interpretability and ability to isolate model performance across different perceptual dimensions. By reporting per-modality accuracy, we provide a fine-grained view of how well the model captures each visual aspect, while overall grounding accuracy reflects the system's general competence in aligning language with perception.

## 6 Results and discussion

### 6.1 Baseline performance (Case 1)

To establish a foundational benchmark, we first evaluate the grounding performance of the baseline model (Case 1), which operates without any syntactic or semantic augmentation. This configuration solely relies on raw sentence descriptions and visual features, namely object color histograms, object centroids for computing spatial relations, and viewpoint feature histograms (VFH). No additional linguistic layer, such as positional indices, part-of-speech tags, or word embeddings, is integrated in this case.

As shown in Figure 6, the baseline model achieves a mean training accuracy of approximately 55.4%, while the corresponding testing accuracy on the original word set (S0) is reduced to 47.7%. Although this evaluation is restricted to the original vocabulary, the noticeable drop in performance between the training and testing sets highlights the model's inability to generalize robustly, even within the observed lexical space. The comparatively large standard deviations values observed across the simulation runs indicate greater prediction error, further highlighting the limited reliability of the learned grounding associations in the absence of structural linguistic cues.

**Figure 6:**
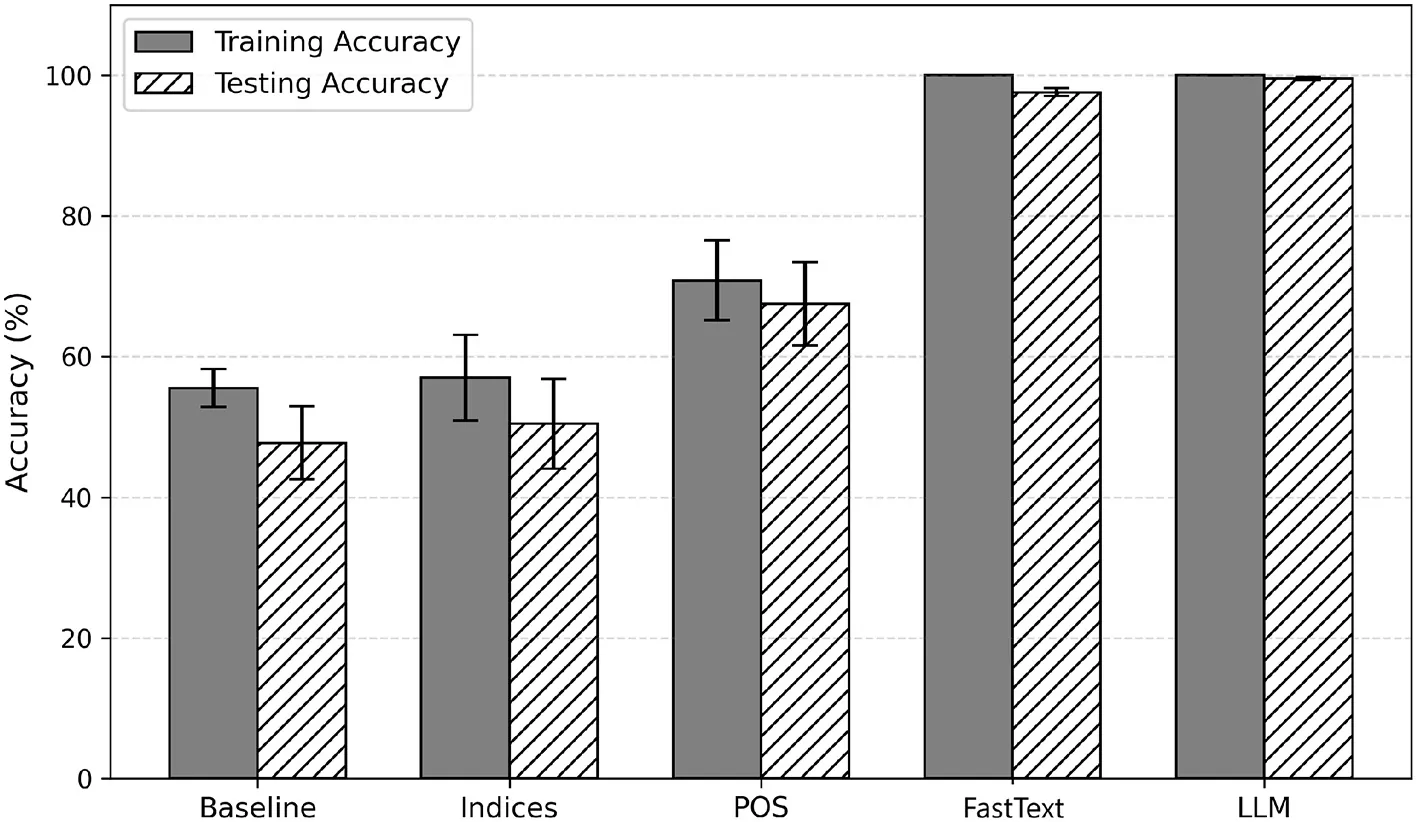
Mean training and testing accuracy across five experimental cases. Error bars indicate the standard deviation across five runs. The baseline model shows limited performance without linguistic augmentation.

A deeper breakdown of sub-accuracy metrics, as presented in Figure 7, reveals modality-specific limitations. The color grounding accuracy is especially poor, averaging only 26.1%, with significant variability. This suggests that the model fails to reliably map color-related words to the corresponding RGB features without additional contextual or syntactic guidance. Object grounding performs slightly better, with an average accuracy of 44.7%, but remains inadequate due to the semantic ambiguity inherent in object references across diverse scenes. In contrast, Spatial Relation grounding achieves a relatively high mean accuracy of 80.8%, likely because the constrained set of relations can be inferred from the structured geometry of dyadic object layouts. Unlike object or color categories, which suffer from high semantic and visual variability, spatial prepositions exhibit consistent centroid displacement patterns that are robust across scenes. Furthermore, the small spatial relation vocabulary minimizes semantic overlap, allowing the baseline model to form stable probabilistic mappings from words to spatial features, even in the absence of syntactic or semantic augmentation.

**Figure 7:**
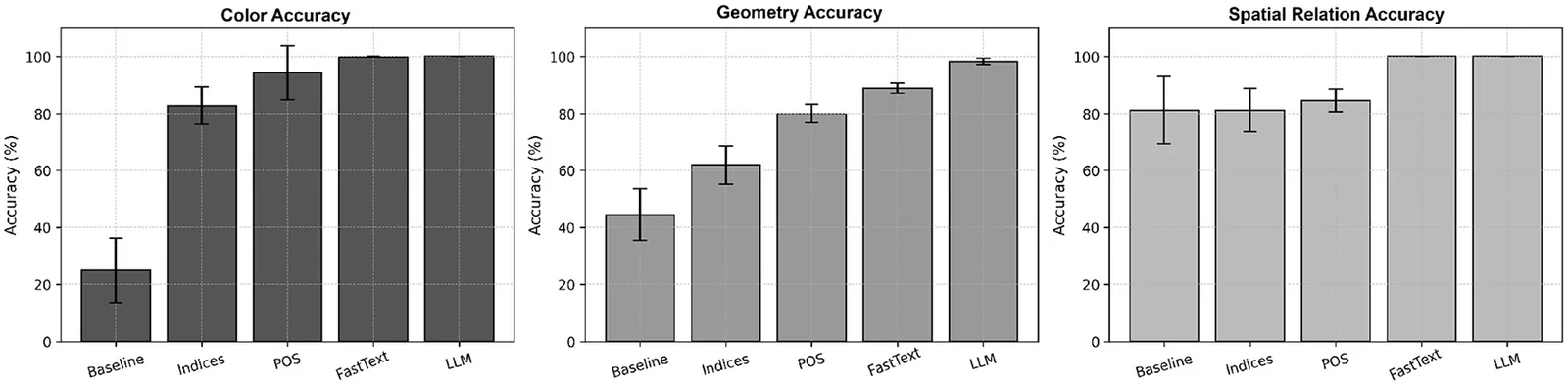
Modality-specific sub-accuracy metrics for Color, Geometry, and Spatial Relation grounding across experimental cases. The baseline model performs poorly in color and object grounding but comparatively better in spatial relations.

These results underscore the limitations of relying solely on multimodal alignment without incorporating any linguistic priors. The baseline case thus serves as a lower-bound reference for assessing the efficacy of syntactic and semantic enhancements in subsequent experimental configurations.

### 6.2 Impact of syntactic layers (Case 2 and Case 3)

To assess the role of syntactic structure in enhancing word grounding, we evaluate Case 2 and Case 3, which incorporate word-level syntactic cues into the generative process. Case 2 introduces *word position indices* to encode positional information, while Case 3 integrates *part-of-speech (POS) tags* as a categorical syntactic layer. These syntactic cues define discrete linguistic states ℓ_*i*_, which index the modality prior π_ℓ_*i*__ and guide the latent modality assignment *m*_*i*_ during inference.

As shown in Figure 6, both syntactic layers yield notable improvements over the baseline. Case 2 achieves an average test accuracy of 50.44%, marking a substantial increase from the baseline's 47.7%. Case 3 further improves this to 67.51%, indicating that POS tags provide a richer syntactic signal than raw word position indices. In both cases, the training and testing accuracy gap is narrowed, and standard deviation error bars reveal improved stability across multiple Gibbs sampling runs.

Figure 7 shows that the addition of syntactic structure significantly benefits object and color grounding. In Case 2, object grounding improves from 44.7% to 61.9%, and color accuracy increases from 26.1% to 82.7%. Case 3 achieves even stronger results, with object accuracy rising to 80.0% and color accuracy reaching 94.2%. These gains reflect the utility of syntactic priors in guiding modality inference: indices help capture positionally relevant patterns (e.g., adjectives often preceding nouns), while POS tags more explicitly distinguish between content-bearing categories (nouns, adjectives, etc.) and functional tokens.

Interestingly, spatial relation grounding remains consistently high and relatively unchanged across all three cases (above 80%), as spatial relations are primarily inferred from centroid-based geometric features. This reaffirms that spatial modality inference is less sensitive to linguistic augmentation and is already well supported by structured visual input.

In summary, syntactic cues—particularly POS tags—enhance the model's ability to disambiguate color and object words by constraining latent modality assignment. These improvements are achieved *without any synonym-based generalization*, as evaluation in Cases 2 and 3 is strictly limited to the original vocabulary (S0). The results emphasize the value of syntactic structure for improving within-distribution grounding accuracy and inference consistency.

### 6.3 Impact of semantic embeddings (Case 4 and Case 5)

To investigate the effect of semantic information in word grounding, we evaluate Case 4 and Case 5, which introduce dense word representations into the fully Bayesian generative model. Case 4 incorporates FastText vectors, which encode subword-level semantics and are robust to morphological variation. Case 5 employs BERT-based embeddings that capture deep contextual semantics derived from large-scale transformer models.

Both cases follow the same Bayesian grounding framework used in the previous experiments, but the role of the linguistic input differs from that in the syntactic configurations. In Cases 4–5, words are represented using dense semantic embeddings rather than discrete word-position or POS-based states. These continuous embeddings do not index a category-specific modality prior π_ℓ_*i*__. Instead, they influence inference through the embedding-aware lexical score ${\tilde{\theta }}_{m,z}\left(\omega_{i}|e_{i}\right)$, which uses semantic similarity between the current word embedding and training-vocabulary embeddings to smooth the modality–cluster word probabilities.

It is important to note that FastText and BERT introduce semantic prior knowledge through large-scale pre-training, whereas word-position indices and POS tags are derived only from the available sentence structure. Therefore, the comparison should be interpreted as evaluating the effect of richer linguistic information within the same Bayesian grounding framework, rather than as a capacity-matched comparison between equally informed models.

As shown in Figure 6, both semantic embedding cases outperform all prior configurations. Case 4 (FastText) achieves a testing accuracy of 97.60% on the original vocabulary set, while Case 5 (BERT) reaches 99.5%, marking the best result across all experiments. The smaller standard deviation across runs also indicates greater stability in the semantic-embedding configurations.

Figure 7 further breaks down the performance across individual modalities. Case 5 shows consistent improvements in color and object grounding, with noticeable gains over FastText. These results indicate that richer semantic representations help the model better associate descriptive words with perceptual evidence, even when the visual scenes are complex or ambiguous. Spatial Relation grounding remains consistently high across all cases due to the strong geometric structure provided by object layouts.

In summary, enriching the model with contextual semantic embeddings significantly enhances both accuracy and stability. These gains are achieved without altering the inference mechanism, highlighting the adaptability of the Bayesian structure to increasingly expressive word representations. The next section evaluates how these embeddings support generalization to unseen lexical variations.

### 6.4 Synonym generalization performance

To assess the capacity of semantic embeddings to enable lexical generalization, we evaluate model performance on synonym-substituted descriptions that preserve semantic intent while introducing novel word forms. While previous results were computed on the original test set (S0), we introduce three additional synonym-augmented versions—S1, S2, and S3—where key object words have been systematically replaced with human-validated synonyms. These substitutions maintain the semantic intent of the sentences while introducing lexical variation not seen during training.

The model does not resolve these substitutions through a mutual-exclusivity or process-of-elimination mechanism. The perceptual-assignment variable *p*_*i*_ is sampled as part of the grounding process and no strict one-to-one word–object matching constraint is imposed. Thus, synonym handling in Cases 4 and 5 is supported by the semantic similarity encoded in the pre-trained embedding space, rather than by a discrete unseen-word probability or a logical elimination rule.

Figure 8 presents the overall testing accuracy across the four sets: the original test set (S0) and three synonym-substituted sets (S1, S2, and S3). These results reflect the model's combined performance across all three grounding modalities: Color, Geometry, and Spatial Relation. While both models begin with strong accuracy on S0 (97.60% for static word vectors, 99.53% for Transformer based Contextual embeddings), performance declines as the lexical substitutions become more complex. By S3, which contains the most challenging synonyms, FastText drops to 83.44%, while BERT maintains a notably higher score of 90.03%. This trend suggests that contextual embeddings are more robust to lexical shifts, likely due to their ability to capture semantic similarity beyond surface forms.

**Figure 8:**
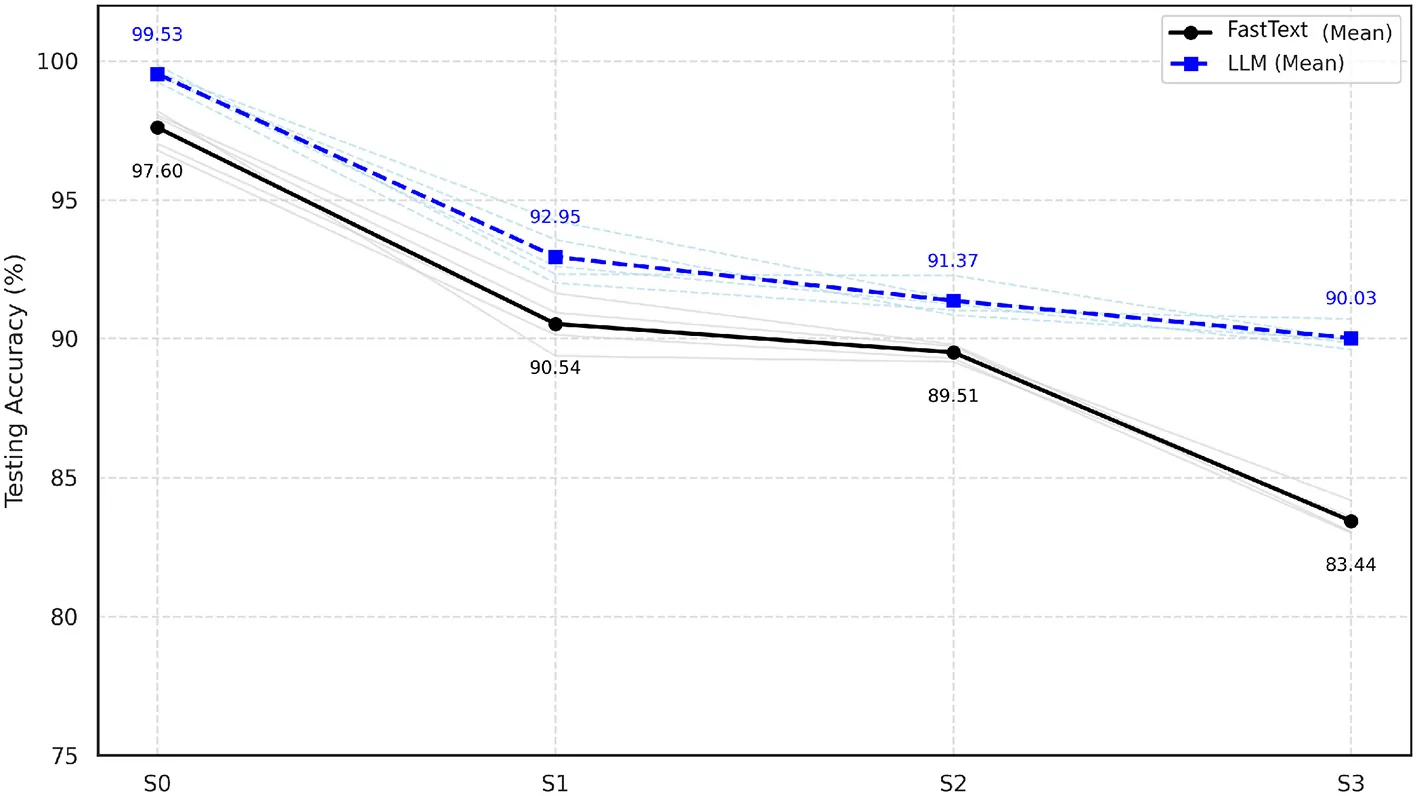
Overall testing accuracy for static word vectors (Case 4) and transformer-based contextual embeddings (Case 5) across the original and synonym-substituted test sets (adapted with permission from Shaukat et al. 2026). S0 denotes the original test set, while S1, S2, and S3 denote synonym-substituted test sets with increasing lexical divergence. The reported accuracy combines performance across all grounding modalities.

Since the synonym substitutions only apply to object words, it is necessary to isolate the model's object grounding performance from the overall scores. Figure 9 reports object-specific accuracy for both models across the same four test sets. Transformer-based contextual embeddings consistently outperform static word vectors, especially as the synonym complexity increases. On S1, BERT achieves 71.74% object accuracy compared to FastText's 63.29%. The margin widens in S3, where FastText degrades to 33.64% while BERT remains at 59.94%. This consistent advantage highlights the BERT model's capacity to align novel synonyms with perceptual features more effectively than FastText, which operates with fixed subword-based representations.

**Figure 9:**
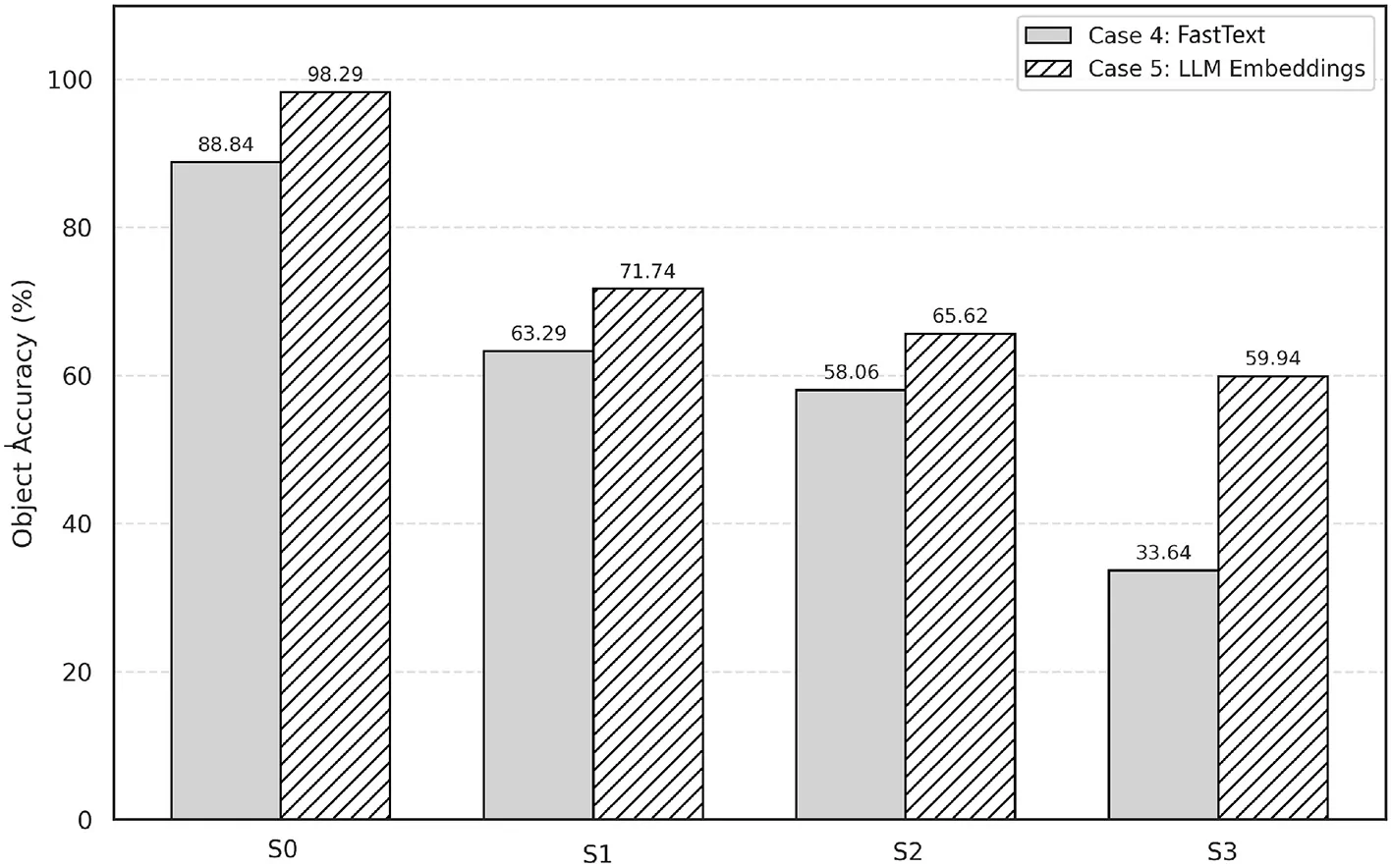
Object-specific grounding accuracy for static word vectors (Case 4) and transformer-based contextual embeddings (Case 5) across the original and synonym-substituted test sets (adapted with permission from Shaukat et al. 2026). S0 denotes the original object words, while S1, S2, and S3 denote synonym-substituted object words with increasing lexical divergence. This figure isolates object accuracy because synonym substitutions were applied only to object-category words.

To evaluate whether the difference between FastText and BERT is statistically reliable under synonym substitution, we performed paired comparisons using the object-wise synonym accuracy values reported in Table 5. Since synonym substitution was applied only to object-category words, the statistical test was conducted across the 19 object categories for each synonym set. BERT achieved higher mean object accuracy than FastText across all three synonym sets. For S1, the mean improvement was 8.45 percentage points, but the difference was not statistically significant (95% CI: –6.28 to 22.86; paired *t*-test, *p* = 0.247). For S2, the mean improvement was 7.56 percentage points and was also not statistically significant (95% CI: –9.83 to 24.91; paired *t*-test, *p* = 0.374). For S3, which contains the most semantically distant substitutions, BERT achieved a statistically significant improvement of 26.30 percentage points over FastText (95% CI: 13.61 to 39.03; paired *t*-test, *p* = 0.00039). These results show that contextual embeddings provide the clearest advantage when object-word substitutions become more indirect and semantically challenging.

**Table 5:** Object-wise grounding accuracy (%) under synonym substitution.

Object	Synonyms 1 (S1)		Synonyms 2 (S2)		Synonyms 3 (S3)	
	FastText	BERT	FastText	BERT	FastText	BERT
Ball	43.3	76.3	30.0	27.4	55.3	51.7
Barrel	46.5	21.8	84.4	1.4	11.2	28.9
Basket	56.7	86.0	72.8	98.0	38.5	100.0
Book	43.7	65.7	61.7	65.6	20.0	52.4
Bottle	52.0	100.0	80.0	31.1	60.8	25.9
Cage	75.3	86.8	44.8	89.1	29.7	62.7
Canister	56.6	89.0	70.6	78.0	25.0	21.0
Car	63.4	96.0	67.2	92.9	27.9	90.5
Cylinder	80.2	100.0	68.7	88.9	22.2	100.0
Cone	68.0	56.5	28.0	54.6	16.0	64.7
Crate	44.9	85.0	18.5	46.8	12.8	50.0
Cube	43.9	50.8	5.5	54.7	19.3	36.5
Cup	89.2	91.2	82.5	100.0	62.3	84.7
Flask	82.7	40.5	58.9	12.3	51.8	63.1
Glass	70.0	35.6	96.0	77.3	56.9	90.1
Knife	69.3	94.5	49.2	70.1	32.6	46.6
Pan	87.5	27.4	64.0	65.2	50.0	60.0
Torus	66.0	67.3	28.7	93.3	36.9	76.6
Toy	63.3	91.2	91.7	100.0	10.0	33.5
**Mean**	**63.29**	**71.74**	**58.06**	**65.62**	**33.64**	**59.94**

Results are reported for three synonym sets (S1–S3) using FastText and BERT embeddings. Values are averaged over five independent runs.

Together, these results demonstrate that semantic embeddings, particularly contextualized ones like BERT, significantly improve robustness to lexical variability in object grounding. While FastText captures basic semantic similarity, it struggles with paraphrastic and ambiguous substitutions. In contrast, BERT maintains grounding accuracy even when encountering object terms not seen during training, reinforcing the benefits of contextual depth and distributional generalization in multimodal grounding systems.

A direct CLIP baseline is not reported because standard CLIP evaluation operates on raw images and text prompts, whereas the proposed model uses segmented object-level descriptors and infers word modalities, object grounding, and referent–landmark spatial relations. Therefore, a CLIP-based comparison would require reformulating the task using rendered object crops, tailored prompts, and separate image-text queries for spatial relations. This would create a different evaluation protocol rather than a like-for-like baseline.

### 6.5 Fine-grained color grounding performance

While Figure 7 reports aggregate color grounding accuracy across all color categories, such averages may mask variability in performance for individual color attributes. To provide a more detailed analysis, Table 6 presents color-wise grounding accuracy for each of the ten color categories across all five experimental configurations. The results demonstrate a consistent improvement from the baseline model to syntactic and semantic variants, indicating that additional linguistic structure substantially enhances color grounding. Semantic embedding-based models achieve near-perfect accuracy across all colors, with the transformer-based contextual embeddings (BERT) yielding uniformly high performance. This suggests that rich semantic representations enable robust alignment between color descriptors and perceptual features, irrespective of color category.

**Table 6:** Color-wise grounding accuracy across experimental cases.

**Color**	Baseline	Indices	POS	FastText	BERT
Blue	0.19	0.72	0.88	0.96	0.99
Brown	0.23	0.80	0.92	0.98	0.99
Cyan	0.23	0.82	0.95	0.98	1.00
Gray	0.28	0.84	0.96	0.99	1.00
Green	0.28	0.85	0.95	0.98	0.99
Orange	0.26	0.83	0.94	0.97	0.99
Purple	0.24	0.81	0.93	0.98	1.00
Red	0.30	0.86	0.97	0.99	1.00
White	0.29	0.84	0.95	0.98	0.99
Yellow	0.31	0.90	0.97	0.99	1.00

Results are averaged over five independent runs.

### 6.6 Fine-grained object grounding performance

While Figures 7, 9 summarize overall object grounding trends, aggregate scores may obscure performance differences across individual object categories with varying geometric complexity. To provide a more detailed analysis, Table 7 reports object-wise grounding accuracy for all object categories across the five experimental configurations. The results reveal a clear and consistent improvement from the baseline to syntactic and semantic variants, with semantic embedding-based models achieving near-ceiling performance across all object categories.

**Table 7:** Object-wise grounding accuracy on original descriptions (S0).

Object	Baseline	Indices	POS	FastText	BERT
Ball	0.52	0.63	0.84	0.98	1.00
Barrel	0.50	0.62	0.82	0.97	0.99
Basket	0.48	0.61	0.81	0.97	0.99
Book	0.30	0.60	0.78	0.96	0.99
Bottle	0.45	0.62	0.80	0.97	0.99
Cage	0.46	0.63	0.81	0.97	0.99
Canister	0.44	0.61	0.80	0.97	0.99
Car	0.49	0.64	0.82	0.98	1.00
Cone	0.32	0.56	0.77	0.95	0.99
Crate	0.48	0.62	0.80	0.97	0.99
Cube	0.41	0.60	0.79	0.96	0.99
Cup	0.47	0.63	0.81	0.97	0.99
Cylinder	0.29	0.55	0.76	0.95	0.99
Flask	0.43	0.61	0.80	0.97	0.99
Glass	0.46	0.64	0.82	0.98	1.00
Knife	0.48	0.63	0.81	0.97	0.99
Pan	0.47	0.62	0.80	0.97	0.99
Torus	0.50	0.64	0.83	0.98	1.00
Toy	0.30	0.58	0.78	0.96	0.99

Results are averaged over five independent runs.

While Figure 9 presents aggregate object-grounding performance under synonym substitution, this summary may conceal variation across individual object categories and levels of lexical divergence. To provide a more detailed perspective, Table 5 reports object-wise grounding accuracy for all object categories across three synonym sets (S1–S3), evaluated using the two semantically enriched configurations (FastText and BERT). All values correspond to mean testing accuracy over five independent runs. The results show that contextual embeddings often improve grounding accuracy under synonym substitution, but this improvement is not consistent across all object categories. In many cases, BERT handles shape-preserving synonyms effectively (e.g., *cup*–*mug, car*–*automobile*), leading to strong grounding performance. However, some substitutions introduce words that are linguistically related but no longer clearly describe the same physical object. In these cases, BERT's embeddings capture real-world semantics—such as brand, function, or typical context—rather than the simple geometric shape used to define CLEVR objects, leading to a mismatch with our geometry-based object representation. This behavior is consistent with prior analyses showing that increased context sensitivity in contextualized embeddings can introduce representation variance and reduce semantic consistency under lexical substitution (Wang and Zhang, 2023). Simpler static embeddings, which encode less contextual information, are sometimes less affected by this mismatch. This fine-grained analysis complements the aggregate trends and shows that while contextual semantic representations generally improve grounding accuracy under synonym substitution, they can also fail when linguistically valid substitutions no longer correspond to the same physical object shape.

Some object-level outliers occur because the synonym sets include substitutions that are linguistically related to the original object label but do not always preserve the same geometric form used by the CLEVR-derived object category. For example, certain S2 or S3 substitutions may reflect a broader category, subtype, brand, material, function, or contextual association rather than a strict shape-preserving synonym. Since object grounding in this framework relies strongly on VFH-based geometry features, such substitutions can reduce object-level accuracy even when the replacement is semantically plausible in language.

## 7 Conclusion

This work presents a fully generative Bayesian framework for multimodal word grounding that systematically incorporates multiple layers of linguistic information. Through five controlled model variants—ranging from a purely perceptual baseline to models augmented with syntactic features and pretrained semantic embeddings—we evaluate how different linguistic layers affect the grounding process. All models are trained using Gibbs sampling, ensuring consistent inference dynamics across configurations.

Experiments conducted on the CLEVR 3D scene dataset show that linguistic inputs significantly enhance grounding accuracy across Color, Geometry, and Spatial Relation modalities. Syntactic features such as word order and part-of-speech tags serve as effective categorical constraints, while semantic embeddings, particularly transformer-based contextual vectors, enable fine-grained distinctions between visually similar objects. The model incorporating large language model embeddings achieves the highest performance, attaining a 53.8% absolute improvement in object grounding accuracy over the baseline on original descriptions.

To assess lexical generalization, we constructed and validated three synonym sets—S1 (close substitutions), S2 (less common), and S3 (semantically distant)—for object-related words. Only the semantically enriched models (Cases 4 and 5) were evaluated under these substitutions. Results show that transformer-based embeddings maintain high accuracy even under substantial lexical variation, outperforming static FastText vectors in both grounding accuracy and semantic consistency. Notably, contextual embeddings show a smaller performance drop across S1–S3, reflecting their capacity to preserve meaning beyond surface forms. The paired statistical comparison between FastText and BERT across the synonym sets is reported in Table 8. The synonym robustness observed in Cases 4–5 reflects lexical transfer enabled by the pre-trained embedding space provided to the model, within the Bayesian multimodal grounding framework. Across all experimental cases, the Bayesian framework provides the common multimodal probabilistic grounding structure through which linguistic inputs are integrated with perceptual evidence and grounding variables are jointly inferred.

**Table 8:** Paired statistical comparison of FastText and BERT object grounding accuracy under synonym substitution.

Synonym set	FastText mean	BERT mean	Difference	*p*-value
S1	63.29	71.74	8.45	0.247
S2	58.06	65.62	7.56	0.374
S3	33.64	59.94	26.30	0.00039

The paired test was performed across the 19 object categories reported in Table 5.

These findings establish a principled, extensible foundation for language-grounding systems, highlighting the complementary roles of syntactic and semantic information in achieving both accuracy and robustness in perceptually grounded language understanding. Building on this word-level foundation, future work can extend the framework toward compositional language understanding in human-robot interaction (Shaukat et al., 2025b).

Although the controlled CLEVR-derived setting enables systematic evaluation of syntactic and semantic effects, it also defines the current scope of the study. Real-world deployment would introduce additional challenges not fully captured in the present experiments, including occlusion, noisy object segmentation, variable illumination, cluttered backgrounds, ambiguous referring expressions, and incomplete visual observations. Since the proposed model relies on object-level visual features such as RGB histograms, VFH descriptors, and centroid-based spatial relations, performance in real-world scenes would depend on the reliability of upstream perception and segmentation modules. This controlled evaluation assumes a known perceptual category structure. In less structured real-world settings, the number of perceptual categories would typically need to be selected or inferred from data rather than specified a priori.

## Data Availability

The raw data supporting the conclusions of this article will be made available by the authors, without undue reservation.

## Footnotes

1 In the present experiments, *K*_*c*_ = 10, *K*_*s*_ = 8, and *K*_*g*_ = 19 were fixed according to the known category structure of the controlled CLEVR-derived dataset.
