# Supplementary material for: The effect of syntactic and semantic information on word grounding through visual perception
Source: Front Artif Intell. 2026 Sep 22;9:1803426. doi: 10.3389/frai.2026.1803426 (PMC13638530; doi:10.3389/frai.2026.1803426)
Supplement: Supplementary file 1 [file Supplementary_File_1.pdf]

Supplementary Material

The Effect of Syntactic and Semantic Information on Word Grounding through Visual Perception

Saima Shaukat, Amir Aly, Anouar Chibani, Thomas Wennekers, and Angelo Cangelosi

Supplementary Appendix A: Reproducibility Details

This appendix provides additional methodological details to improve the reproducibility of the experimental setup. It describes the synthetic 3D scene-generation procedure, the validation of scene descriptions, visual feature extraction, Bayesian model settings, experimental configurations, and synonym-set construction and validation protocol.

A.1 Synthetic 3D Scene Generation

The dataset was generated using a customized CLEVR-style 3D scene-generation pipeline implemented through Blender's Python API. The final experimental dataset consisted of 240 synthetic 3D scenes. Each scene contained 3–4 segmented objects and was paired with a manually authored natural-language description.

A.1.2 Scene-Generation Procedure

The scene-generation procedure used in the study can be summarized as follows:

1. Select 3–4 objects from the predefined object set and assign colors from the predefined color set.
2. Assign object size, material, orientation, and position, and construct one or more spatial configurations using the predefined spatial relation set.
3. Apply generation constraints to preserve distinguishability, physical plausibility, visibility, and interpretable spatial relations.
4. Render the scene in Blender and validate the result for visibility, object/color distinguishability, spatial consistency, and absence of rendering artifacts.
5. If validation fails, adjust the scene configuration and re-render until the constraints are satisfied.
6. Export object metadata and object-level point clouds, then extract RGB histograms, VFH descriptors, centroid coordinates, and centroid-difference features.
7. Create a manually authored natural-language description covering the relevant object, color, and spatial-relation terms.

Each generated scene was paired with a natural-language description covering the relevant object, color, and spatial-relation terms needed for the grounding task. Each description was checked against the corresponding rendered scene and corrected if it contained mismatched object names, incorrect color references, missing objects, or spatial relations inconsistent with the scene layout.

A.2 Bayesian Model Settings

The Bayesian grounding model associates each word with a perceptual modality and a corresponding perceptual assignment: an object index for Color or Geometry, an ordered referent–landmark pair of object indices for Spatial Relation, or $\varnothing$ for Others.

The three perceptual modalities are Color, Geometry, and Spatial Relation. In the present experiments, we used $K_c = 10$, $K_s = 8$, and $K_g = 19$, corresponding to the predefined Color, Spatial Relation, and object-category sets used in the dataset.

The model uses categorical distributions with Dirichlet priors for modality assignment, word distributions, and cluster priors, and Gaussian distributions with Normal–Inverse–Wishart priors for continuous visual features. The hyperparameters used in the Bayesian model were $\lambda = 1.0$, $\delta = 0.01$, $\gamma = 0.035$, $\alpha_c = 0.30$, $\alpha_s = 0.35$, and $\alpha_g = 0.30$.

For the embedding-aware lexical score (Case 4 and Case 5), the semantic mixing coefficient was fixed at $\rho = 0.70$, and the softmax temperature was fixed at $\tau = 0.10$.

For the Normal–Inverse–Wishart priors, we used $\beta = (\kappa_0, \mu_0, \Lambda_0, \nu_0)$, where $\kappa_0 = 10^{-3}$, $\mu_0 = 0_d$, $\Lambda_0 = 0.01 I_d$, and $\nu_0 = d + 2$, with $d = 15$, $d = 3$, and $d = 308$ for Color, Spatial Relation, and Geometry features, respectively, where I_d denotes the $d \times d$ identity matrix.

A.3 Synonym Set Construction

The synonym sets were constructed to evaluate whether the model could generalize to object-word substitutions not observed during training. Only object-category words were replaced. Color terms, spatial-relation terms, and functional words were kept unchanged. This design ensures that any change in generalization performance can be attributed specifically to object-word substitution.

Three synonym sets were constructed:

- **S1: Direct lexical equivalents.** These substitutions are close lexical alternatives to the original object word. Examples include "cup" → "mug" and "car" → "automobile".
- **S2: Less direct but semantically plausible substitutions.** These substitutions are related to the original object word but may be broader, narrower, or less direct. Examples include "cup" → "teacup" and "car" → "vehicle".
- **S3: Indirect or context-dependent associations.** These substitutions are semantically related but more distant, requiring deeper semantic association. Examples include "cup" → "coffee" and "car" → "Audi".

The synonym words were introduced only during testing for the semantic embedding configurations. They were not included in the training descriptions.

Table A2 provides examples of synonym substitutions used in the synonym-set construction process.

Table A2: Examples of object-word substitutions used in the synonym validation process.

**Original object**	**Synonym-set level**	**Example substitute**
car	S1	automobile
cup	S1	mug
car	S2	vehicle
cup	S2	teacup
book	S2	textbook
car	S3	Audi
book	S3	Bible
canister	S3	Coke

A.4 Annotator Guidelines for Synonym Validation

Three independent human annotators validated the synonym sets. Annotators were asked to judge whether each proposed substitute was semantically plausible for the corresponding original object word and whether it matched the intended synonym-set level.

The validation focused on the following criteria:

- **Semantic plausibility:** the substitute should be meaningfully related to the original object word.
- **Set-level consistency:** the substitute should match the intended level of lexical distance:
  - S1 should contain direct lexical equivalents.
  - S2 should contain less direct but still semantically plausible substitutions.
  - S3 should contain indirect or context-dependent semantic associations.
- **Visual interpretability:** the substitute should remain interpretable in relation to the object category used in the generated scene.
- **Avoidance of unrelated substitutions:** candidates that changed the object category entirely or referred primarily to a color, material, action, or spatial relation were not retained.

Final synonym selections were made based on majority agreement and semantic plausibility. Inter-rater agreement was computed using Fleiss' kappa and achieved a score of 0.90, indicating strong annotation consistency.

The semantic proximity of representative object–synonym pairs was also evaluated using cosine similarity in FastText and BERT embedding spaces. These similarity scores were used to support the analysis of synonym generalization and to compare the behavior of static and contextual semantic representations.
